# Integration of biological avatars and digital twins for “ex vivo clinical trials”

**DOI:** 10.1016/j.ebiom.2026.106467

**Published:** 2026-09-01

**Authors:** Charles L. Howe

**Affiliations:** a Human Model Systems Core, Mayo Clinic, Rochester, Minnesota, USA; b Division of Experimental Neurology, Mayo Clinic, Rochester, Minnesota, USA

**Keywords:** Biological avatars, Organoids, Organ-on-a-chip, Digital twins, Precision medicine, Drug development

## Abstract

Drug development is slow, costly, and prone to late-stage failure, in part because animal models poorly predict human responses. Two human-relevant technologies are maturing in parallel: biological avatars, defined as patient- or stem-cell-derived models such as organoids and organ-on-a-chip systems, and digital twins, defined as computational models that integrate a patient’s molecular and clinical data to forecast treatment responses. We propose the ex vivo clinical trial concept, in which an avatar and a digital twin are coupled in an iterative loop so that laboratory measurements refine the computational prediction and the prediction guides the next experiment, allowing candidate therapies to be tested and prioritised before a patient is exposed. We review the platforms, their predictive performance in cancer, cystic fibrosis, and liver toxicity, the conditions under which they fail, and the qualification, turnaround, and standardisation requirements that must be met before such trials can inform drug development or clinical care.

## Introduction

The development and clinical testing of new drugs are hindered by numerous obstacles, including high attrition rates for therapies that have passed preclinical testing, lengthy R&D timelines, and scientific and ethical concerns stemming from extensive reliance on nonhuman animal models.^1^^,^^2^ Despite annual expenditures in excess of $30 B USD for preclinical drug development and more than $300 B USD for total pharmaceutical R&D,^3^^,^^4^ the success rate for taking a drug from phase I testing to FDA-approved clinical implementation remains below 10%,^5^ with some therapeutic areas, such as drugs for cardiovascular and nervous system diseases, having a probability of launch from phase I that is less than 5%.^2^ In an analysis of 174 phase II/III failures for which a cause was reported, 132 (76%) were attributed to lack of efficacy or safety concerns.^6^ For example, TGN1412 administered to human volunteers at 0.2% of the safe dose identified in preclinical nonhuman animal models resulted in life-threatening cytokine storms in all 6 subjects within 90 min of delivery of the first dose,^7^ whereas BIA-102474 treatment induced brain haemorrhage, necrosis, and death during the first-in-human clinical trial, despite extensive preclinical testing.^8^ The use of human “biological avatars” for preclinical and clinical testing may overcome these challenges^9^ (see Table 1 for definitions of terminology used throughout this review). These avatars include adult stem cell-derived organoids,^10^ which retain the tissue-specific genetic, epigenetic, and functional features of organs such as the intestine or liver, and induced pluripotent stem cell (iPSC)-derived organoids,^11^ which can be differentiated into a broader array of tissue types that can model more complex disease states.^12^ The FDA Modernisation Act of 2022^13^ removed the mandate requiring nonhuman animal testing for drugs before proceeding to clinical trials,^14^ thereby enabling the potential use of human stem cell-derived organoids and microphysiological systems for assessing drug safety and efficacy. This regulatory shift has now opened the door to the adoption of biological avatars into both preclinical R&D pipelines and “clinical trials in a dish”.^15^

**Table 1:** Terminology.

Term	Definition	Key distinction or relationship
Biological avatar	A living, patient- or stem-cell-derived model (organoid, organ-on-a-chip, microphysiological system or bioprinted tissue) that replicates human physiology while capturing individual genetic diversity and disease-specific phenotype.	Umbrella term for the wet, biological half of an ex vivo clinical trial; paired with the dry, computational digital twin.
Organoid	An in vitro, self-assembled, three-dimensional micro-tissue derived from stem cells that recapitulates tissue or organ micro-anatomy and functionality.	The biological building block; may be static or integrated into a device.
Microphysiological system (MPS)	A fit-for-purpose device containing one or more engineered organs or organ substructures in a controllable, real-time-monitored microenvironment, which may incorporate organoid cell formations.	Superset that adds engineered control (flow, mechanics, sensing) to organoid biology.
Organ-on-a-chip	A subset of MPS that replicates the in vivo dynamics, functionality, structure and pathophysiological response of a single organ system.	A single-organ MPS.
Body-, disease- or human-on-a-chip	Interconnected organ mimics with continuous or recirculating flow operating in physiologically scaled regimes.	A multi-organ MPS supporting systemic pharmacokinetic and pharmacodynamic analysis.
Digital model	A computational representation of a physical system with no automated data exchange; any updating is manual.	Lowest integration; does not react to the physical system.
Digital shadow	A computational representation automatically updated one-way from the physical system, without closed-loop influence back on it.	Adds automated physical-to-virtual flow; still not bidirectional.
Digital twin	A dynamically updated, predictive computational system with decision relevance and bidirectional interaction between the virtual and physical systems.	Highest integration; virtual and physical update each other.
Biomedical digital twin	A computable, personalised replica of a biological system (cell, organ or patient), dynamically updated with data and used to simulate counterfactual interventions for hypothesis testing, response prediction and decision-making.	Application of the digital twin concept to biology; the computational half of an ex vivo clinical trial.
Counterfactual	A twin-based estimate of the outcome the physical system would exhibit under an action that was not actually taken, with all other variables and the underlying physiology held fixed.	The quantity a digital twin predicts, for example the response to an untested dose.
Co-clinical study	Testing in a patient-derived or animal model run in parallel with the matched human trial, correlating model and patient outcomes.	Parallel testing that lacks a closed, bidirectional avatar-twin computational feedback loop.
Ex vivo clinical trial	The iterative, bidirectional coupling of a biological avatar and a patient- or population-level digital twin to predict, refine and prioritise therapy before or alongside in-human testing.	The integrated framework proposed here; extends co-clinical testing by adding the computational feedback loop.

Definitions, key distinctions, and relationships among the principal terms used throughout this Review. The digital model/shadow/twin gradation is defined by the direction of automated data flow between the physical and virtual systems.

Parallel to the development of these cellular models, computational “digital twins” that integrate large and heterogeneous datasets, collected across different scales and modalities, ranging from genomic profiles to lifestyle behaviours, are being used to provide mechanistic, in silico representations of patients.^16^ By modelling complex, interconnected systems such as gene regulatory pathways, cellular signalling networks, and organ-level interactions, digital twins can be used to predict therapeutic responses and to simulate disease progression. This multiscale approach accommodates the inherent complexity of adaptive biological processes and facilitates the fine-tuning of interventions for personalised treatment plans. Digital twins transcend traditional data-driven approaches by incorporating causal insights that are rooted in network theory and systems biology, thereby providing a mechanistic basis for evaluating different drug treatment regimens and minimising reliance on traditional experimental methods. As with the regulatory shift in support of biological avatars, the 2022 FDA Modernisation Act has also paved the way for the use of computational models, artificial intelligence, and machine learning for drug development.^17^

**Fig. 1:**
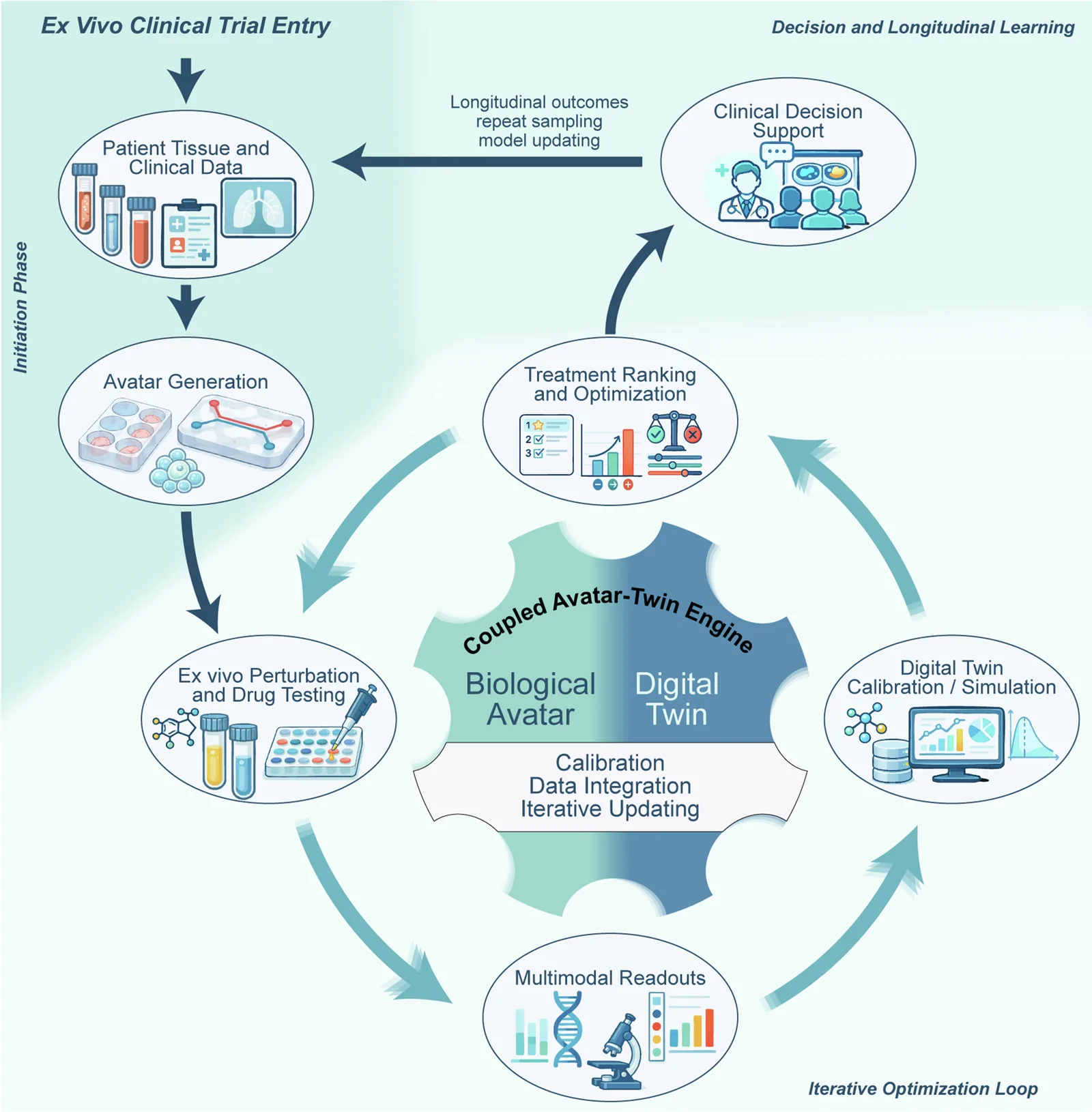
**Ex vivo clinical trial framework integrating biological avatars and digital twins.** Illustration of a conceptual workflow for an ex vivo clinical trial system that links patient-derived biological avatars with computational models in an iterative decision-support framework. The process begins with collection of patient tissue that is used to generate a patient-relevant biological avatar, such as organoids or microphysiological systems. These avatars are subjected to ex vivo perturbation and drug testing, and the resulting multimodal readouts are used to calibrate and iteratively update a digital twin built around the patient’s multimodal data. The coupled avatar-twin engine supports digital twin calibration and simulation, followed by treatment ranking and optimisation and ultimately clinical decision support. A longitudinal feedback loop returns clinical outcomes, repeats sampling, and updates patient data back into the system, enabling iterative learning over time. This approach emphasises that ex vivo clinical trials are not a single assay, but a coupled wet-dry translational workflow in which biological experimentation and computational modelling inform one another through repeated calibration, data integration, and updating. The schematic illustrates the patient-level implementation of this loop. At the population level, the same coupled avatar-twin approach can be applied across qualified reference panels to support candidate ranking, dose or trial design, and toxicity assessment.

On the basis of these developments, we propose that “ex vivo clinical trials”, built around the integration of biological avatars and computational twins, may accelerate and help catalyse the development of safer and more effective therapies by enabling the iterative refinement of therapeutic candidates in a tightly controlled environment that reflects true human biology (Fig. 1). In this context, the biological avatars serve as living laboratories that capture patient-specific genetic diversity, disease manifestations, and environmental factors and the digital models integrate and refine these insights to drive improvements in the therapeutic agent and to forecast how the therapy performs in larger or more varied patient populations. Dynamic feedback loops between the avatars and computational models have the potential to be particularly catalytic, with real-time data streams generated from organoids refining computational predictions, and these refined models in turn being used to guide new hypotheses, speed up lead selection, and inform the design of subsequent in vivo clinical trials. In this manner, ex vivo clinical trials may reduce costly late-stage therapy failures and prevent adverse events. These trials may also enhance the development of precision treatments tuned to the unique biology of each patient and could help mitigate the ethical and scientific limitations imposed by traditional animal research.

## Biological avatars

### Human-relevant experimental platforms

ASTM International^18^ defines an organoid as “an in vitro, self-assembled, 3D micro-tissue or organ developed from stem cells that recapitulates tissue or organ micro-anatomy and functionality”. ASTM further defines microphysiological systems as “fit-for-purpose devices, containing one or more engineered organs [or] organ substructures … in a controllable microenvironment” that can be “monitored under real-time” and may be composed of “organoid cell formations”. Organ-on-a-chip devices represent a subset of microphysiological systems that replicate the “in vivo dynamics, functionality, structure, and/or (patho)physiological response” of an organ system. Likewise, “body-on-a-chip”, “disease-on-a-chip”, and “human-on-a-chip” microphysiological systems represent “interconnected organ mimics [organoids] with continuous or recirculating flow” operating in scaling regimes that are consistent with physiologically based pharmacokinetic models that use measurements of absorption, exposure, metabolism, and excretion and that are composed of patient-derived and stem cell-derived biological materials integrated into a nonbiological platform. Together, these systems are biological avatars that serve to replicate true human physiology while capturing genetic diversity and disease-specific phenotypic characteristics.

**Fig. 2:**
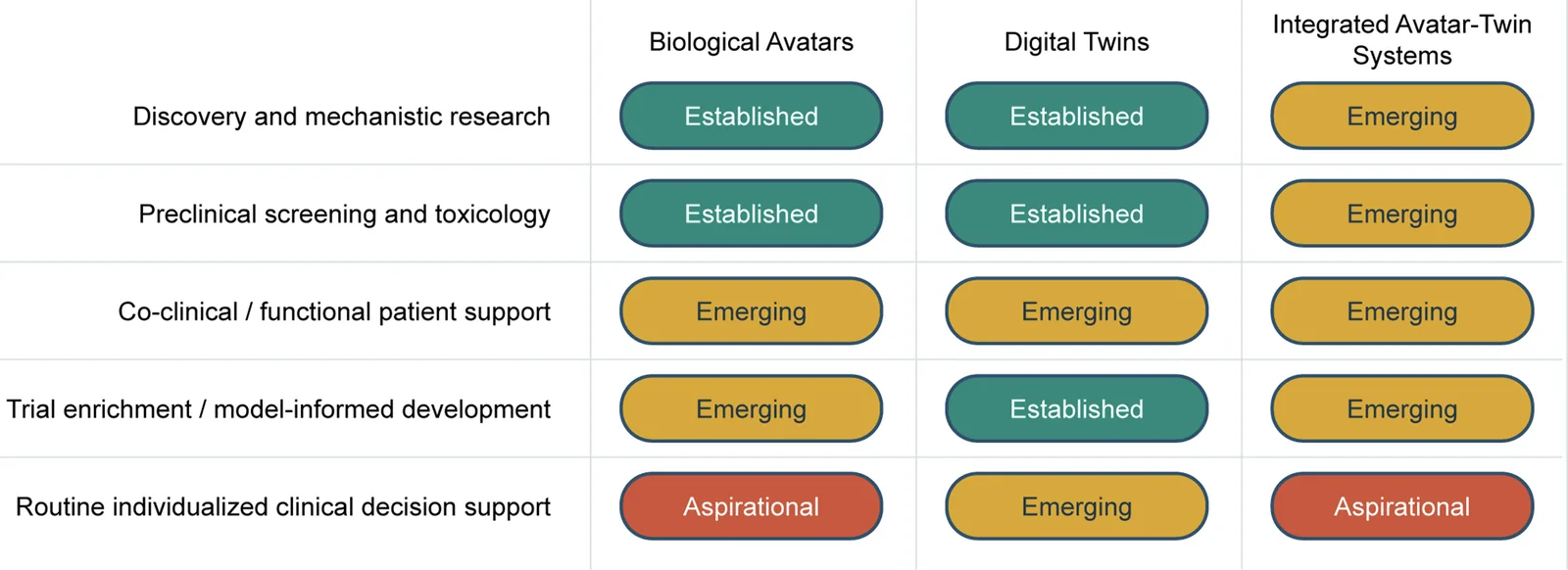
**Translational maturity landscape for biological avatars, digital twins, and integrated avatar-twin systems.** Summary of the relative translational maturity of biological avatars, computational systems, and integrated avatar-twin frameworks across major domains of application, ranging from discovery research to routine individualised clinical decision support. Biological avatars are comparatively mature for discovery and preclinical screening but remain less mature for routine patient-level decision support. The computational column is labelled “digital twins” for simplicity, but here the term is used broadly to encompass digital models, digital shadows, and digital twins, which differ in the degree of coupling, iterative updating, and decision relevance. Within this broader computational category, model-informed development is relatively mature, whereas patient-specific, dynamically updated decision-support systems remain less established. Integrated avatar-twin systems are currently best viewed as an emerging translational framework, with the greatest promise in bounded use cases such as co-clinical support and model-informed development, but these systems remain aspirational for routine individualised clinical care. Maturity should be interpreted as context dependent, reflecting the specific context of use, target population, assay or decision window, validation requirements, operational feasibility, and consequences of error.

The development of organoid-based systems has continued to accelerate, with self-organising 3D aggregates of polarised cortical neuroepithelium derived from embryonic stem cells reported in 2008,^19^ self-organising intestinal organoids from adult tissue-derived stem cells described in 2009,^20^ and iPSC-derived organoids described in 2011.^21^ By 2018, Nature Methods named organoids the “Method of the Year 2017”.^22^ As of 2025, adult tissue-derived stem cell-based organoids have been generated from more than 20 tissue types, ranging from the intestine, liver, and lung to the placenta, prostate, and vagina and from nearly 20 tumour types.^23^ In parallel, induced pluripotent stem cells have been used to generate organoids for many of the same adult stem cell-derived endodermal derivatives, but also for many nonepithelial cell-based tissues, including ectodermal and mesodermal derivatives that are not readily generated from tissue-derived stem cells, such as brain, skin, heart, and kidney cells. However, a central caveat in the translation of these systems is that static epithelial organoids, vascularised organ-on-chip platforms, immune-competent tumour microenvironment systems, and linked multi-organ microphysiological systems represent a spectrum of models that differ substantially in biological completeness, controllability, reproducibility, readout richness, and clinical decision readiness.^9^^,^^23^ Based on this, the most useful way to view the application of biological avatars is as a maturity ladder: organoids and microphysiological systems as established research tools, digital twins as emerging tools for guiding therapy decisions, and fully integrated, bidirectionally updated coupled avatar-twin systems used to guide first-line therapy decisions as an aspirational state (Fig. 2).^23^^,^^24^

### Adult tissue-derived organoids

Organoids derived from stem cells harvested from adult tissues collected by biopsy or surgical resection recapitulate key aspects of the architecture and function of the mature human organ. For example, multipotent Lgr5-positive stem cells isolated from biopsied intestinal crypts readily reconstitute the polarised epithelial structure, maturity, and functionality found in the intact intestine when embedded in extracellular matrix and cultured in the presence of tissue-specific growth factors.^20^ The addition of species- and organ-specific signalling agonists and antagonists prevents growth arrest in these organoids, permitting essentially unlimited proliferation while maintaining genomic stability.^25^ However, despite serving as a fast and efficient method for generating patient-specific avatars that exhibit barrier function and nutrient absorption, adult stem cell-derived epithelial organoids cannot capture the entire cellular repertoire present in the donor organ and therefore cannot fully recapitulate the complex function of the organ.^26^ Coculturing adult stem cell-derived epithelial organoids with primary immune and stromal cells is one strategy to reconstitute some of the multicellularity of the organ. For example, the addition of immortalised stomach mesenchymal cells to gastric epithelial organoids promoted a broader repertoire of cell types,^27^ while coculturing intestinal organoids with pericryptal fibroblasts and neurons derived from the myenteric plexus enhanced growth and maturation.^28^

### Induced pluripotent stem cell-derived organoids

In contrast to adult stem cell-derived organoids, iPSC-derived organoids are generated from adult somatic cells that have been reprogrammed into an embryonic-like stemness state via the expression of pluripotency factors^29^ or exposure to chemical epigenetic modifiers.^30^ These stem cells are then pushed to acquire different downstream fates along the ectodermal, endodermal, or mesodermal lineage via genetic manipulation,^31^ defined growth and trophic factor cocktails,^21^ substrate modifications,^32^ and small-molecule signalling modulators.^33^ Downstream lineage cells can also be induced from adult somatic cells via direct reprogramming or transdifferentiation, bypassing the pluripotency stage.^34^ Based on this capacity, induced stem cell-derived organoids are able to incorporate not only epithelial cells, but also mesenchymal, stromal, and parenchymal cell types. For example, iPSC-derived intestinal organoids generated by sequential growth factor manipulations following endoderm induction and hindgut specification develop a polarised, columnar epithelium with villus-like structures containing crypt-like proliferative regions that are integrated with smooth muscle cells, subepithelial myofibroblasts, and fibroblasts.^21^ Similarly, iPSC-derived embryoid bodies grown under conditions that promote ectodermal differentiation develop rosettes that contain neural progenitor cells.^35^ Following neuroepithelial expansion, these progenitor cells can be pushed to enter neurogenic and gliogenic phases and to subsequently mature into brain region-specific cell types and structures.^36^ Different iPSC-derived brain regions can be fused together in assembloids^37^ to facilitate brain-like interconnectivity. In parallel, nonneural cells such as microglia and brain vascular endothelial cells can be generated from other iPSC-derived progenitor populations and mixed into neural organoids to recapitulate neuroimmune interactions^38^ and neurovascular coupling.^39^

### Building a biological avatar

The construction of a biological avatar appropriate for ex vivo clinical trials requires a quality-managed, fit-for-purpose pipeline that begins with sourcing and patient consent, selection of optimal quality control-validated biological materials, standardised and reproducibly staged derivation and patterning, and context-of-use qualification of the avatar for testing. The starting material may be adult stem cells and/or primary cells isolated from patient biopsies and surgical resections, or iPSCs generated through the reprogramming of somatic cells.^10^^,^^11^ The nature of the reprogramming method used for generating iPSCs is less critical than in the cell replacement space, but the reprogrammed material must meet stringent metrics for subject identity (short tandem repeat profile matching^40^), genomic stability (karyotyping, copy number variation sequencing), pluripotency, trilineage potential, and sterility.^41^ The microenvironment should favour xeno-free and chemically defined conditions and the use of tunable matrices or hydrogels to standardise stiffness, ligand density, and viscoelasticity.^42^ Directed differentiation should proceed through defined stages (for example, definitive endoderm, regional fate specification, maturation) that have clearly identified milestones and phenotypic and/or functional metrics, such as surface marker expression, barrier integrity, metabolic competence, and electrophysiological profile.^43^ These metrics also serve as explicit go/no-go release criteria that functionally align wet lab qualifications with the data needs of the downstream digital twin, ensuring that the avatars are competent and optimised for the intended readouts.

The avatars may function as a single-organ model that focuses on specific organ functions (e.g., hepatic metabolism in liver-on-chip systems) or as a multi-organ platform that integrates several organs to study systemic interactions.^44^ Genome editing approaches, in which CRISPR/Cas9 technology is used to introduce specific disease-associated mutations into otherwise healthy iPSCs or to correct mutations in patient-derived cells, increase the sophistication and physiological relevance of biological avatars.^45^ Likewise, microbiome integration via the incorporation of commensal and pathogenic microorganisms enables studies of host-microbiome interactions in drug metabolism and inflammatory conditions.^46^ Finally, the inclusion of modules for fluid perfusion and vascularisation (via self-assembled endothelium, bioprinted endothelium, or prepatterned microchannels) facilitates physiological oxygenation, metabolic clearance, and fluid flow shear stress.^39^ However, given the increased QC burden and reproducibility risk that comes with added complexity, the goal should be to develop an avatar platform that is sufficiently complex for the context-of-use but no more complex than necessary.^9^^,^^24^

**Fig. 3:**
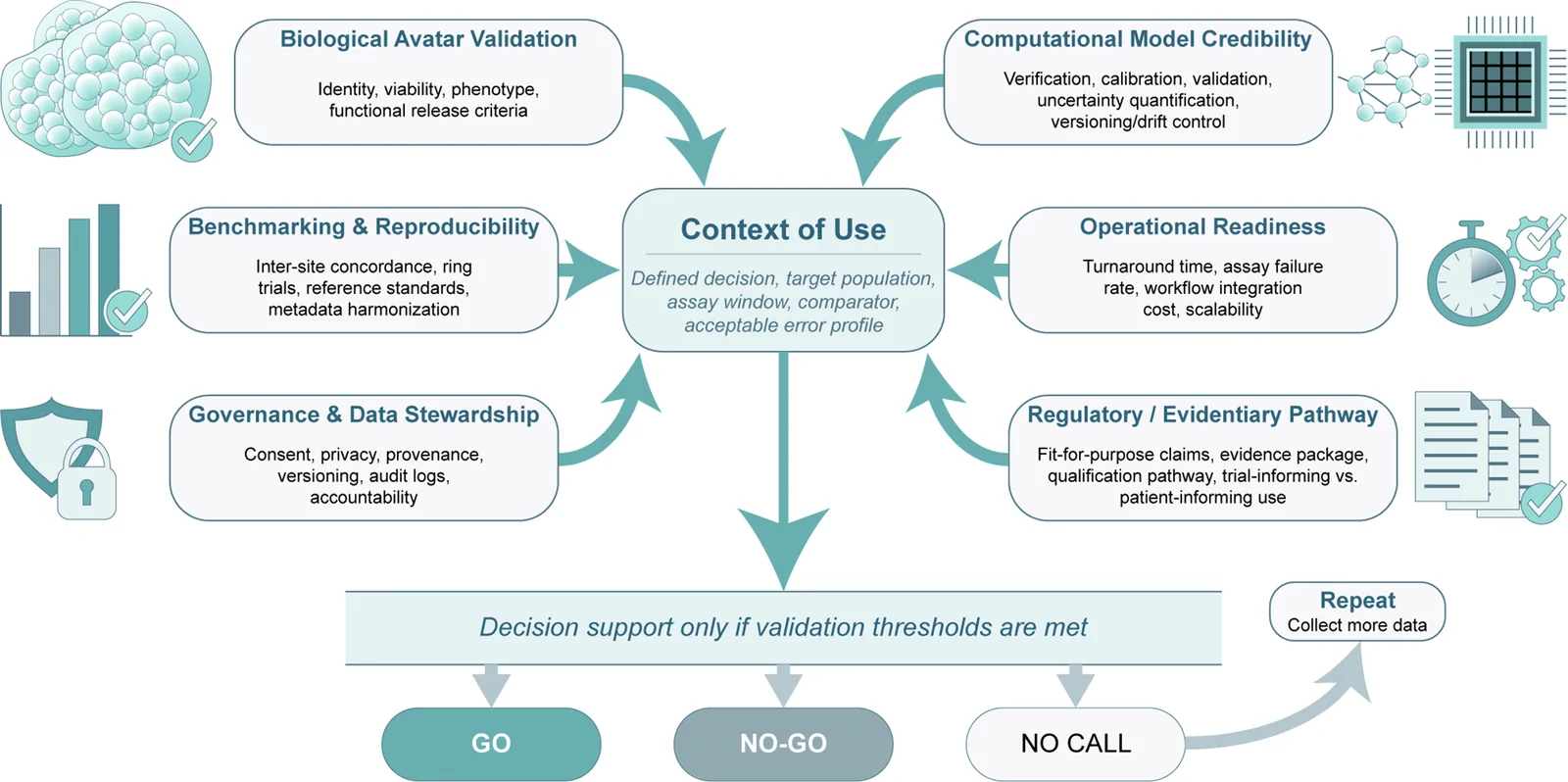
**Validation, benchmarking, and governance framework for ex vivo clinical trial systems.** Illustration of a fit-for-purpose framework for evaluating biological avatar and computational systems intended to support ex vivo clinical trials. At the centre is context of use, which defines the intended decision, target population, assay window, comparator, and acceptable error profile. Around this core, six interdependent domains determine whether a system is sufficiently credible for translational use: biological avatar analytical validation, computational model credibility, benchmarking and reproducibility, operational readiness, governance and data stewardship, and regulatory/evidentiary pathway. Together, these domains emphasise that translational readiness is not established by biological sophistication or predictive performance alone, but by a broader evidentiary framework that includes reproducibility, workflow feasibility, uncertainty management, provenance, accountability, and fit-for-purpose qualification. Decision support should be considered only when validation thresholds are met, and possible outputs include go, no-go, and no-call/insufficient confidence, the latter acknowledging that a system may appropriately defer when the available evidence is inadequate for a reliable recommendation.

To ensure reproducibility and scalability,^47^ living biobanks and versioned lots of cells and organoids need to be established with documented provenance and release records^25^ and the final “release to experiment” will depend upon explicit context-of-use and performance criteria, such as cellular identity and/or purity, functionality, time window of sustained function, and material compatibility.^48^^,^^49^ In addition to standardised biobanks and versioned lots, protocol provenance, batch tracking, and pre-specified inclusion/exclusion criteria will be fundamental to the successful application of biological avatars. It is equally important to recognise that single-site success is insufficient to support translational claims. Inter-site benchmarking, harmonised metadata capture, and ring-trial-style reproducibility studies will be required for avatar-based support of clinical and/or regulatory decisions.^50^ Ultimately, the question is not whether an avatar is good, per se, but whether it is fit for a bounded decision in a defined population and timeframe. For example, an avatar used to rank drug regimens in a metastatic cancer model within a two-week window has a different evidentiary burden than an avatar used to infer chronic toxicity risk or to guide first-line therapy selection. For translational credibility, each context-of-use should specify the population, specimen source, assay window, readout, decision consequence, comparator, and acceptable error profile.^9^^,^^49^ Thus, “qualification” of the biological avatar becomes an evidence package built on the interdependent analytical, computational, governance, and regulatory requirements of the use case (Fig. 3).

### Biofabrication and device integration

Higher-order structure and physiological control can be engineered into avatars through biofabrication and microfluidic device integration. Bioprinting positions cells and matrix into organ-like architectures, while microfluidic chips impose perfusion, shear stress, and air-liquid interfaces that static culture cannot; the methods, bioink and matrix chemistries, and representative organ-on-chip platforms are summarised in Supplementary Table S1. This added control carries added risk. The clearest example is polydimethylsiloxane (PDMS), the elastomer from which most organ-on-chip devices are built: its transparency and gas permeability are useful, but it absorbs hydrophobic small molecules and thereby distorts drug exposure and pharmacokinetic readouts,^51^ which can require coatings, alternative materials such as thermoplastics or glass, or materials-aware computational correction. As with the biological components of an avatar, reproducibility depends on benchmarking fabrication, material properties, recirculation, oxygenation, and sensor calibration.^9^^,^^49^

### Clinically relevant biological avatars

**Fig. 4:**
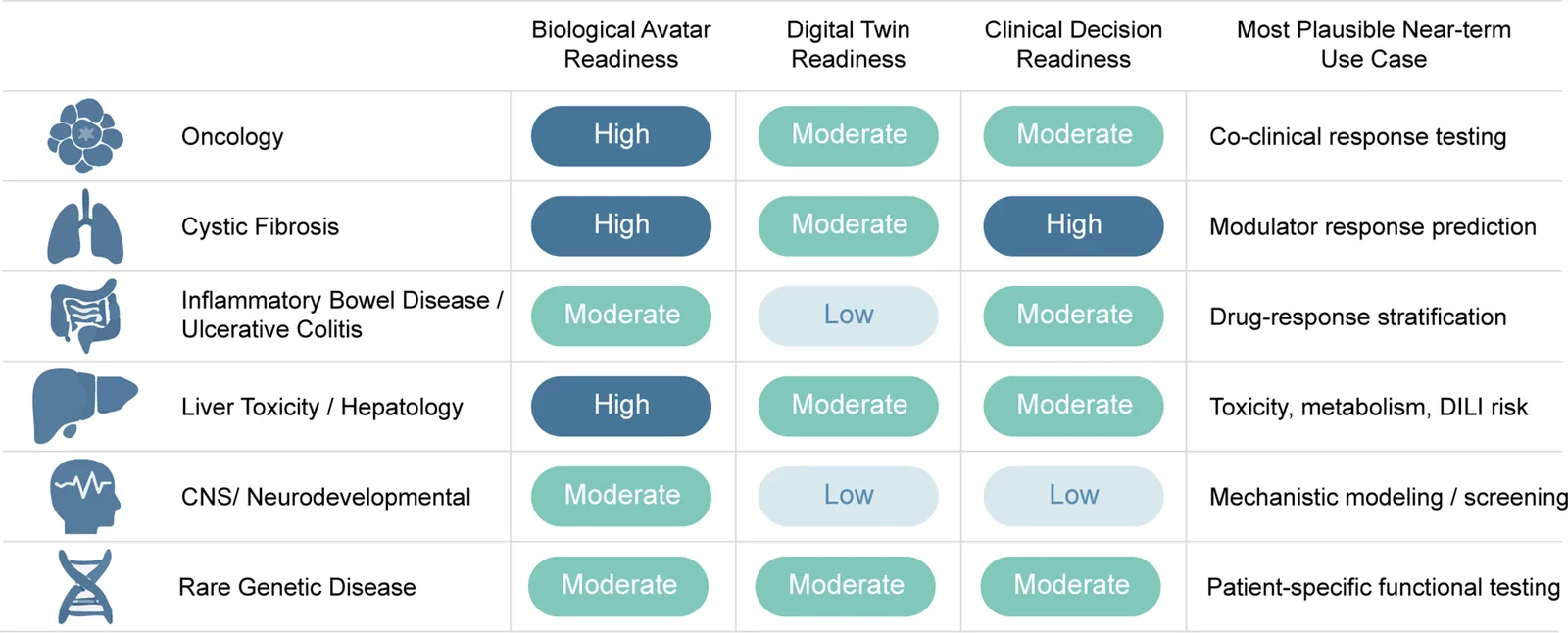
**Disease-specific use-case matrix for biological avatars and digital twins in ex vivo clinical trials.** Summary of the relative readiness of biological avatars, computational systems, and clinical decision applications across selected disease domains relevant to ex vivo clinical trials. Biological avatar readiness, digital model/twin readiness, and clinical decision readiness are shown for oncology, cystic fibrosis, inflammatory bowel disease/ulcerative colitis, liver toxicity/hepatology, CNS/neurodevelopmental disease, and rare genetic diseases, together with the most plausible near-term use case in each setting. The computational column refers broadly to digital models, digital shadows, and digital twins, recognising that these differ in coupling, iterative updating, and decision relevance. The matrix highlights that readiness is disease specific and context dependent: oncology, cystic fibrosis, and liver-focused applications currently show some of the strongest translational potential, whereas CNS applications remain more limited and are best suited to specific mechanistic or screening contexts. Across all domains, near-term opportunities are strongest where biological relevance, assay tractability, turnaround time, and validation requirements are aligned to a clearly defined use case.

Across organ systems, biological avatars have moved well beyond proof of concept.^23^ Liver, intestinal, and lung platforms reproduce organ-level drug responses with sufficient fidelity to recapitulate clinical toxicities and treatment effects, tumour-microenvironment models can outperform conventional xenografts for some immunotherapy predictions, and fluidically linked multi-organ systems capture interorgan metabolism and systemic pharmacokinetics that single-organ formats cannot. Collectively, these examples indicate that avatars are most predictive where the readout is mechanistically tied to the disease process and the assay window matches the clinical question, and least mature where vascularisation, immune competence, or a functional barrier is essential, as in most brain models (Fig. 4). The platform-by-platform examples, the specific drugs and processes modelled, and their references are provided in Supplementary Table S2. A head-to-head comparison of the principal avatar platforms, contrasting their advantages, limitations, and suitability for ex vivo clinical trials, is provided in Supplementary Table S3. In brief, adult stem cell-derived organoids are strongest for well-defined epithelial decisions such as colorectal therapy selection, organ-on-chip platforms are strongest where flow or barrier physiology drives the readout, and multi-organ systems best capture systemic questions but remain research-grade rather than decision-grade at this time. The recurring constraint is not whether a single avatar performs well, but whether that performance generalises across batches, sites, and patient populations.

## Digital twins

### Computational models of human physiology

The National Academies of Sciences, Engineering, and Medicine define a digital twin as “a set of virtual information constructs that mimics the structure, context, and behaviour of a natural, engineered, or social system (or system-of-systems), is dynamically updated with data from its physical twin, has a predictive capability, and informs decisions that realise value”.^52^ The definition further stipulates that “the bidirectional interaction between the virtual and the physical is central to the digital twin”. Unfortunately, the term digital twin is frequently applied to models that are, in fact, static mechanistic models or to models that are unidirectional. The term has also been applied to retrospective cohort generators and other models that do not capture the strongest aspects of a digital twin.^53^ More precise terminology distinguishes a digital model, which is a computational representation that is not dynamically updated by the physical system, from a digital shadow, which is updated by the physical system but does not drive iterative intervention within a closed loop. A true digital twin is a dynamically updated predictive system with decision relevance and bidirectional interaction with the physical system. In the biomedical context, a digital twin is a computable replica of a biological system that is personalised, dynamically updated with data, and used to simulate counterfactual interventions for hypothesis testing, response prediction, and decision-making. In this context, a counterfactual is the twin-based estimate of the outcome the physical system would exhibit in response to an action or sequence of actions that were not actually taken while all other variables and the underlying physiology of the system remain fixed. For example, in a cellular digital twin, the counterfactual is the forecast of the cell state if a certain transcription factor is knocked out or if a different combination of drug, dose, or treatment time is applied.

Given that the implementation and regulatory taxonomy regarding biomedical digital twins is still evolving, we identify three key elements of twin design: 1) representational level (molecule, cell, network (organoid), organ, organism, population); 2) learning paradigm (mechanistic, data-driven, hybrid); and 3) output modality (function monitoring, simulation, and decision-making). Within the context of ex vivo clinical trials, we more narrowly define a digital twin as an in silico framework that represents an organ (or organoid), a patient, or a population using a model based on multiscale and multimodal input data to predict therapy responses and guide therapy decisions.^16^ At the tissue level, digital twins may integrate multiomics data (genomic, epigenomic, proteomic, spatial), biomechanical parameters (fluid dynamics), and drug-specific properties (solubility, protein binding) to model drug pharmacokinetics, pharmacodynamics, and systemic effects. At the patient level, by capturing and modelling the complex, adaptive nature of biological systems, digital twins can facilitate drug efficacy testing, predict adverse effects, and guide refinement of dosing regimens for specific populations or individuals. Critically, digital twins benefit from interplay between mechanistic and data-driven approaches, with hypothesis-driven generative modelling providing causal insight and advanced AI and network analysis enabling learning from large, heterogeneous datasets.

### Building a digital twin

A comprehensive digital twin for drug development encompasses multiple interconnected components that collectively simulate complex biological processes. A physiologically based pharmacokinetic (PBPK) model that simulates drug absorption, distribution, metabolism, and excretion across multiple organ compartments often forms the core of such digital twins.^54^ Overlaying demographic data (height, weight, age, sex), physiological parameters (hepatic blood flow, glomerular filtration rate), and enzyme/transporter phenotypes (such as CYP1A2 activity) on the PBPK model enables individualisation of the “virtual patient” with realistic exposure distributions.^55^ Layering a pharmacodynamic (PD) component onto the PBPK framework by linking drug concentrations to biological effects^55^ improves dose-response prediction and therapeutic index estimation. For chronic conditions, disease progression submodels capture the natural history against which treatment effects are inferred, enabling prediction of long-term treatment effects. Interindividual variability modules within the PBPK model quantify the combined influence of genetic, physiological, and environmental factors that contribute to differences in drug responses. Wearable and mobile sensors can also provide continuous or near-continuous real-world physiological and behavioural data between clinical encounters, allowing the twin to capture disease and treatment response dynamics outside the clinic.^56^ Incorporating patient-specific data into these models significantly improves accuracy - for example, adding the CYP1A2 phenotype to a caffeine PBPK model significantly increased the percentage of accurate predictions.^57^ Likewise, incorporating drug-drug interactions into PBPK models within the context of known metabolic pathways and transporter interactions improves prediction of potential adverse interactions. Hybridisation with machine learning components allows continuous refinement of the digital twin as new data accumulate, improving accuracy and predictive power. Finally, where appropriate, coupling PBPK/PD layers to quantitative systems pharmacology disease modules can provide mechanism-of-action insights that can be used to create credible “what if” scenarios for trials.

Computational modelling of organ-on-chip devices using platforms such as COMSOL Multiphysics enables optimisation of the architecture, materials, laminar fluid flow, and mechanical forces prior to fabrication, reducing prototyping costs, shortening development timelines, and improving device fidelity. For example, simulating a precision-cut liver slice in a microfluidic device identified design modifications that tripled the amount of viable material that could be maintained within the actual device,^58^ while simulations of a lung-on-a-chip device optimised the design for analysis of nanoparticle deposition on lung epithelial cells.^59^ Simulation of laminar flow, concentration gradients, thermal diffusivity, and molecular transport can enhance device design to improve oxygen transport and optimise heat and mass transfer at interfaces.^60^ Such models also provide estimates of drug and nutrient transport across membranes, shear stress effects on cells, and peristaltic motion-induced mechanical stresses.

Digital twins require rigorous calibration using experimental data, beginning with a data integration process that harmonises diverse data types, including time-course measurements of drug concentrations, metabolite formation, biomarker responses, and molecular profiles. Calibration is the primary mechanism by which a model is tuned to reflect the real-world system, especially when the underlying models rely on simplified parameters. For example, the DigiLoCS framework^61^ integrates experimental time-course data measuring drug depletion in a liver-on-chip system with compound-specific parameters such as lipophilicity and molecular weight and hardware parameters such as flow rate and architecture. Nelder-Mead optimisation^62^ was used to align experimental measurements with simulated drug concentrations within each compartment of a three-compartment physiological model and Sobol sensitivity analysis^63^ was used to identify the permeability of the endothelial compartment, surface area of the liver interstitium, and intrinsic on-chip clearance rate as critical calibration parameters for the twin. This approach was validated by predicting the on-chip clearance of 32 different compounds using the digital twin of the liver-on-chip and extrapolation to in vivo clearance in human subjects.

### Computational advances in digital twin technology

Digital twins integrate models across biological scales. Molecular dynamics simulations predict drug-target binding and off-target events, agent-based models capture emergent behaviour in heterogeneous cell populations,^64^ finite-element transport models predict drug penetration and concentration gradients in tissue,^65^ and physiologically based pharmacokinetic and pharmacodynamic models, now routine in regulatory submissions, integrate these into whole-body exposure and response predictions.^66^ Hybrid approaches that couple mechanistic models with machine-learning components retain interpretability while improving accuracy, for example in predicting drug-drug interactions.^67^ For clinical use, calibrated uncertainty quantification using Bayesian or ensemble methods^68^ and explainability techniques such as SHapley Additive exPlanations^69^ will be essential, since a prediction without a credible confidence bound cannot support a treatment decision.

Generative and agentic methods turn the twin from a static simulator into an active component of the avatar-twin loop. Generative models synthesise tissue-scale morphology and simulate perturbation-induced phenotypes in silico before wet-lab work, and generative digital twins map molecular profiles to morphology to support in silico perturbation.^70^ These models depend on the spatial-omics and multiplexed-imaging platforms inventoried in Supplementary Table S4, and the generative architectures and agentic frameworks listed in Supplementary Table S5. Agentic twins close the loop by combining active learning with mechanistic constraints to choose the next dose, schedule, or combination that most reduces predictive uncertainty within safety bounds,^71^ running virtual experiments on agent-based simulators such as PhysiCell and PhysiBoSS^72^^,^^73^ and updating both the twin and the underlying model as real measurements accumulate.

## Ex vivo clinical trials

### Concept and methodology

The ex vivo clinical trial concept (Fig. 1) bridges conventional in vitro testing and in vivo trials. Within this concept, there are two distinct use cases with different evidentiary requirements and timelines. The first, and the one with nearer-term use, is population-level support for drug development and regulatory decision-making, in which avatar-twin systems could rank candidates, refine dose or trial design, and flag toxicity against qualified reference panels. The second, longer-horizon use, is individualised clinical decision support, in which a patient-specific avatar and digital twin are used to select or modify treatment for that individual. The latter requires patient-specific fidelity, turnaround within the clinical decision window, prospective validation against individual outcomes, and clinical governance beyond that needed for population-level model-informed development. These applications therefore occupy different positions on the translational maturity landscape (Fig. 2). Either use case could be pursued through two possible implementations.

In the first implementation, an avatar-based trial runs in parallel with in-human testing by (1) acquiring fresh patient material (e.g., tissue biopsy or blood collection for stem cell isolation or reprogramming), (2) building patient-specific organoids or organ-on-chip systems, (3) exposing the biological avatar to the same treatments and schedules used clinically in the matched patient, (4) correlating avatar responses with clinical outcomes, and (5) updating predictive models to inform future decisions. This parallelisation yields earlier signals of likely success or failure and enables proactive therapy switches when the avatar indicates non-responsiveness. This approach offers several advantages over current drug trials, including real-time feedback on treatment efficacy before clinical progression is evident, the opportunity to test alternative therapies when primary treatment shows a poor response, and accumulation of matched avatar-patient datasets to improve future predictive models.

In the second implementation, a pre-existing patient-matched biological avatar (perhaps drawn from a bank of genetically or phenotypically similar organoids) is paired with a digital twin of that patient, and real-time responses in the biological material are incorporated back into the digital model to identify the optimal therapeutic approach. Once identified, the selected treatment is trialled in the patient, potentially using the parallelisation strategy of the first implementation. The advantage of this approach is that the patient is not directly treated until the therapy has been tested in the coupled biological avatar-digital twin system, greatly reducing safety issues and financial burden arising from a failed therapeutic response. While efforts to date have focused almost exclusively on cancer^74^^,^^75^ (Supplementary Table S6), there is great potential to apply these approaches both to first-in-human drug trials and to the identification of optimised therapy and polytherapy approaches to treat serious and complex human diseases. How the integrated avatar-twin ex vivo clinical trial differs from conventional animal-to-human translation, static avatar screening, and digital-only in silico simulation, across human relevance, iterative wet-dry feedback, patient-specific resolution, and the basis for moving to in-human testing, is summarised in Supplementary Table S7. The central contrast is that only the integrated avatar-twin approach couples bidirectional wet-dry feedback with patient-specific resolution. Conventional animal-to-human translation lacks human relevance and feedback, static avatar screening lacks model coupling and uncertainty propagation, and digital-only simulation lacks a living system to correct it (Supplementary Table S7).

### Qualification and decision criteria

For an ex vivo clinical trial to inform an in-human decision, the avatar-twin system must clear a prespecified qualification bar defined for the specific context of use, not a single universal standard. We propose that each context of use prespecify (i) the decision to be supported and the population, specimen source, and assay window in which it applies; (ii) the comparator against which performance is judged; (iii) a minimum analytical performance, expressed as the sensitivity, specificity, and calibration of the coupled prediction against the clinical endpoint; and (iv) the acceptable error profile, recognising that a false negative and a false positive carry different costs. The field has not agreed on numerical thresholds for these criteria; what exists are demonstrated performance figures that can serve as provisional anchors. Illustrative qualification panels for three representative use cases, specifying the avatar platform, reference panel, primary readout and metric, demonstrated performance, and principal current limitation for each, are provided in Supplementary Table S8. Applied to a concrete case, the four criteria specify a drug-induced liver injury panel as follows: 1) the *decision* is whether to flag a candidate as hepatotoxic before first-in-human dosing (population, small molecules entering safety testing; specimen, a hepatocyte-based liver-on-a-chip; window, the multi-day exposure over which albumin, ALT, and morphological injury are read out); 2) the *comparator* is the IQ Microphysiological Systems reference set of blinded toxic and non-toxic drugs; 3) the *analytical target* is sensitivity and specificity against that reference set; and 4) the *error profile* weights a missed hepatotoxin above a falsely flagged safe compound. With these specifications, a liver-on-a-chip reached 87% sensitivity and 100% specificity across 27 blinded drugs, meeting the consortium qualification guidelines for that context of use,^76^ whereas the same platform would not clear a bar written for idiosyncratic injury, which it is not designed to detect. In colorectal cancer, pooled organoid testing predicted standard-of-care response with a positive predictive value near 68% and a negative predictive value near 78% against a 47% baseline response rate,^77^ and prospective testing against 5-fluorouracil and oxaliplatin has reported an area under the receiver-operating curve of 0.78–0.88 using a prespecified sensitivity cutoff.^78^ These benchmarks define what is currently achievable; they do not yet constitute a regulatory standard, and establishing context-specific qualification thresholds remains an open requirement (Supplementary Table S8).

Because the avatar and the twin are distinct measurement systems, they will sometimes disagree, and a credible system must prespecify how disagreement is adjudicated rather than resolving it post hoc. We propose a tiered rule. Where the avatar readout and the twin prediction agree within the prespecified confidence interval, the concordant call proceeds. Where they disagree to a clinically meaningful degree, the system defaults to the more conservative call with respect to patient safety and triggers a defined escalation: a repeat assay on an independent avatar lot, interrogation of avatar quality-control metrics and twin calibration residuals to localise the source of discordance, and, if discordance persists, referral to multidisciplinary review rather than an automated recommendation. A disagreement is clinically meaningful when it would change the recommended action - that is, when the avatar readout and the twin prediction fall on opposite sides of the prespecified decision boundary, rather than differing only within the calibrated confidence interval. What counts as the more conservative call is itself context specific and must be prespecified: in toxicity screening this means flagging possible harm and withholding or deprioritising the compound, whereas in a patient with late-stage cancer and limited treatment options, the more conservative action may be to retain a candidate, since withholding a potentially active therapy also carries risk. The basis for the disagreement, the escalation steps, and the final adjudication should be documented within the evidence package supporting any regulatory submission, converting discordance from a hidden failure mode into an auditable decision step. Because a repeat assay on an independent lot may not fit within the clinical decision window, the escalation path needs to include a time-bounded fallback: if turnaround time precludes re-testing, the system returns a no-call and defers to standard care rather than forcing a low-confidence recommendation.

The system includes an explicit no-call output for cases in which neither a positive nor a negative recommendation is warranted. A no-call should be triggered by prespecified conditions: failure of the avatar to meet release criteria, such as inadequate organoid viability, poor formation efficiency, or low barrier integrity; failure of the twin to meet a minimum calibration standard in held-out validation; or predictive uncertainty exceeding the prespecified bound for the decision at hand. A no-call is a safeguard rather than a failure; it signals that the avatar-twin assessment has not produced information sufficient to override standard care, and it prevents low-confidence outputs from being mistaken for actionable predictions. Reporting no-call rates alongside sensitivity and specificity is necessary for clinicians, sponsors, and payers to judge real-world utility (Fig. 3).

The strongest evidence for avatar-based treatment selection comes from oncology and cystic fibrosis. In colorectal cancer, patient-derived tumour organoids have predicted irinotecan response, chemoradiation outcomes, and progression-free survival across multiple cohorts,^74^^,^^75^^,^^77^^,^^79^ and a prospective phase 2 trial of organoid-guided therapy selection reported that 17 of 34 patients met the progression-free-survival endpoint at two months.^80^ For context, genome-targeted therapy was estimated to benefit 7% of US patients with cancer in 2020.^81^ In cystic fibrosis, rectal-biopsy organoids accurately predicted individual responses to CFTR modulators including ivacaftor and lumacaftor,^82^ the HIT-CF programme used organoid screening to prioritise modulator therapies across 489 patients,^83^ and organoid testing correctly predicted the clinical failure of ataluren before the phase III trial readout.^84^ Study-by-study results, predictive performance metrics, and additional non-cancer use cases are provided in Supplementary Table S6.

### Regulatory considerations

For the nearer-term population-level use case, the regulatory environment for ex vivo trials is continuously evolving (Fig. 3). Potential pathways to implementing such trials include the use of companion diagnostics^85^ based on avatar responses to include or exclude patients, the development of adaptive trial designs that prospectively incorporate avatar-derived biomarkers to guide treatment adjustments,^86^ and hybrid trial endpoints that combine traditional measures with outputs from the biological avatar-digital twin system. The 2024 acceptance of a letter of intent for an organ-on-a-chip system to screen for drug-induced liver injury^87^ through the Innovative Science and Technology Approaches for New Drugs (ISTAND) pilot program,^88^ and the 2026 acceptance of a letter of intent for an in silico AI-driven digital liver model for the same indication,^89^ mark key milestones for ex vivo clinical trial development. Multi-stakeholder initiatives such as the IQ MPS Working Group^49^ and the NCATS Tissue Chip^48^ are developing performance criteria, reference compounds, and reporting standards that will support trial submissions. These regulatory changes are based on the growing recognition that biological avatars have tremendous potential to improve clinical trial efficiency and outcomes,^15^ particularly for complex diseases with high interpatient variability.

### Cost efficiency

The estimated cost of bringing a new drug to market ranges from approximately $1–$4 billion.^90^ However, this number underrepresents the total costs incurred during the development and testing of many drugs. For example, it is estimated that $42.5 billion was spent on clinical-stage Alzheimer’s disease drug testing between 1995 and 2021, yet only 6 drugs were FDA-approved for Alzheimer’s disease by 2021,^91^ four of which share the same mechanism-of-action. Between 2002 and 2012, 413 Alzheimer’s disease clinical trials evaluated 244 unique compounds, and after excluding 14 compounds still in phase 3 at the time of analysis, only 1 of 230 evaluable compounds was approved (229 of 230 failed).^92^ Most of these drugs failed due to the absence of confirmed clinical effects relative to placebo or due to unacceptable toxicity. Both of these outcomes may be greatly reduced by ex vivo clinical trial testing, with earlier identification of nonviable candidates freeing up resources to focus on compounds with a higher probability of success.

### Accelerated timelines

Conventional drug development currently requires more than 10 years from initiation of phase I trials to regulatory review and approval.^93^ The application of integrated ex vivo clinical trials using biological avatars and digital twins could accelerate this timeline by enabling parallel testing of doses, regimens, and trial designs to shift risk earlier, refine patient selection for in vivo trials, and provide virtual bioequivalence^94^ that could supplement or replace specific trial elements. Ex vivo clinical trials may also accelerate prioritisation of specific drug candidates, structural variants, formulations, and combinations relative to traditional nonhuman animal model-based testing.

### Enhanced predictivity

The poor translation of nonhuman animal model-based preclinical testing to human clinical outcomes is a major challenge for drug development.^95^ In addition to the inherent scientific limitations of extrapolating human responses from nonhuman models,^7^ the historical reliance on animal testing, especially research involving nonhuman primates and other large animal models, is fraught with ethical concerns.^96^ Replacement of preclinical animal models with human-relevant biological avatars and biologically informed digital twins that are calibrated to human data could reduce animal model usage and increase the accuracy of predicting clinical drug responses. These models may also avoid the bias that arises when clinical trials are performed in homogeneous populations that do not reflect the diversity of the eventual patient population. Ex vivo clinical trials could also facilitate testing in populations that are traditionally excluded from clinical trials, such as pregnant women, children, older adults, and patients with complex multimodal comorbidities.

### Turnaround time, cost, and limitations

Operational feasibility depends on returning a result within the clinical decision window. In colorectal cancer, organoid chemograms testing a panel of 25 agents have been delivered with a median turnaround of six weeks (range four to ten) from biopsy, with an establishment rate of approximately 62% (8 of 13) from core needle biopsies.^97^ Once organoids are established, the drug-sensitivity assay itself runs in roughly one to two weeks. Microfluidic micro-organosphere formats can shorten this interval further: a droplet-microfluidic platform generated patient-derived micro-organospheres from low-volume biopsy tissue and returned a colorectal cancer drug-response readout within 14 days of biopsy.^98^ Turnaround is workflow-dependent and can be the principal constraint for the most aggressive tumours, yet it is not invariably prohibitive: an optimised patient-derived glioblastoma-organoid platform identified candidate treatments within 13–15 days of tumour resection in a proof-of-concept series,^99^ whereas approaches that require extensive ex vivo expansion can exceed the window in which a treatment decision must be made. While the macro-level economic case rests on attrition costs, as described above, published per-patient cost and cost-effectiveness data remain sparse; organoid assays are less costly than animal models but more costly than cell-line screens, and the case for routine adoption will depend on cost-effectiveness evidence that does not yet exist.

These results should be considered against their limitations. Much of the evidence is concentrated in oncology and comes from small cohorts, many analysed retrospectively, in which organoid responses were compared with outcomes after treatment rather than used to assign it; such designs model rather than prospectively predict response. The prospective experience has been mixed and instructive. In the single-arm SENSOR trial, only 6 of 61 enrolled patients ultimately received organoid-guided targeted therapy, the remainder were lost mostly because of clinical deterioration during standard of care or the absence of an actionable in vitro hit, and none of the six treated patients showed a durable clinical response, underscoring both the logistical attrition and the gap between ex vivo signal and in vivo benefit in heavily pretreated disease.^100^ More recent prospective data are more encouraging: in a larger metastatic colorectal cancer study, organoid drug-sensitivity screens for 5-fluorouracil and oxaliplatin predicted patient response with positive and negative predictive values near 0.8 and were associated with progression-free and overall survival.^78^ The divergence between these results points less to a binary verdict on the technology than to the critical role of cohort selection, timing within the treatment course, and assay design.

Performance is, accordingly, assay- and context-dependent. The same colorectal approach that predicted response to irinotecan-based therapy failed to predict outcome for 5-fluorouracil plus oxaliplatin^74^; subsequent optimisation traced part of this discordance to screening-medium composition, with removal of the antioxidant N-acetylcysteine markedly improving the correlation between organoid and clinical oxaliplatin response.^101^ In cystic fibrosis, organoid swelling correlates with CFTR function at the genotype level and is highly repeatable within subjects, yet a ceiling effect at high modulator response limits discrimination among strong responders: in a uniform F508del-homozygous cohort the magnitude of in vitro swelling did not predict the extent of individual clinical improvement on elexacaftor/tezacaftor/ivacaftor,^102^ and in a multicentre study the highest and lowest organoid responders did not differ in individual clinical outcomes even though organoid response magnitude remained associated with sweat-chloride and ppFEV1 change at the pooled level.^103^ In drug-induced liver injury, current liver-on-a-chip models capture intrinsic, concentration-dependent toxicity but are not designed to capture idiosyncratic injury, which depends on adaptive immune components these models currently lack.^76^ The recurring lesson is that an avatar is fit for a bounded decision rather than globally valid, and batch-to-batch and inter-site variability can move a borderline readout below a decision boundary. Closing that reproducibility gap through harmonised protocols, reference standards, and ring-trial benchmarking is the field’s central near-term task.

## Conclusions

Biological avatars, built as adult- and iPSC-derived organoids and organ-on-chip systems and paired with mechanistic, data-driven digital twins, offer a human-relevant testbed that addresses three chronic barriers to drug development: poor clinical translatability, long timelines, and the ethical and scientific limits of animal models. In an ex vivo clinical trial, the avatar generates high-resolution readouts of exposure, efficacy, and toxicity in a physiological microenvironment, while the paired twin integrates those signals with the patient’s multiomics, clinical, and demographic data to forecast response. Iterating between the wet avatar and the dry twin tightens uncertainty bounds and supports the identification of doses and regimens before a patient is exposed, improving the odds of success while reducing risk.

Population-level support for drug development and regulatory decision-making is likely to be deployable sooner than routine individualised clinical decision support, though a plausible longer-horizon clinical operating model can also be envisioned. In that longer-horizon model, at a clinical decision point, a patient-specific avatar is established or retrieved from a biobank, and a continuously updated twin, calibrated to the avatar readouts, ingests electronic health record, imaging, wearable sensor data, and multiomics data. Avatar-twin-ranked treatments are reviewed by a multidisciplinary board in the context of standard guidelines, and where uncertainty remains, rapid-cycle avatar experiments adjudicate among the leading candidates before therapy begins, all within a governance structure ensuring privacy, provenance, model transparency, drift monitoring, and audit trails. Reaching that state will require coordinated progress on five fronts: reproducible avatars with consensus protocols and release criteria; calibrated, explainable twins with versioned parameters and uncertainty quantification that meet regulatory expectations; explicit context-of-use qualification that names the decision, the acceptable error profile, and the validation plan; interoperability through common data schemas and curated reference datasets; and automation that links real-time sensing, liquid handling, and experiment planning within clinically meaningful turnaround times.

Looking further ahead, the avatars themselves will become more dynamic, with embedded sensing of oxygen, pH, and metabolites, closed-loop feedback, and the integration of biological rhythms turning periodic snapshots into continuous data streams that strengthen real-time twin calibration. As spatial omics mature and generative twins model temporal disease trajectories, discovery, preclinical evaluation, trial design, and clinical care may eventually be connected by a single avatar-twin backbone, allowing clinicians to pretest therapies on a patient’s biological and digital surrogates before administering them in vivo. Pursued systematically, this approach could shorten development, reduce the ethical burden of animal research, and bring genuinely precision-tailored therapeutics within reach.

### Outstanding questions

• What context-specific sensitivity, specificity, and calibration thresholds should a coupled avatar-twin system meet before it can inform an in-human treatment decision, and who should define them?
• How should discordance between an avatar readout and a twin prediction be adjudicated, and how should that adjudication be documented in a regulatory submission?
• What minimal qualification panels and reference standards are required for the major use cases, such as liver toxicity, colorectal cancer therapy selection, and cystic fibrosis modulator testing?
• What are the real per-patient costs, turnaround times, and cost-effectiveness of avatar-twin testing relative to existing genomic or functional assays?
• Can inter-site reproducibility be demonstrated through ring trials at a level sufficient to support multicentre clinical and regulatory use?

**Search strategy and selection criteria**

References for this Review were identified through searches of PubMed, Web of Science, and Scopus, and through the reference lists of identified articles. The search combined terms for biological avatars (“organoid”, “patient-derived organoid”, “tumour organoid”, “organ-on-a-chip”, “microphysiological system”, “bioprinting”), for computational models (“digital twin”, “physiologically based pharmacokinetic model”, “quantitative systems pharmacology”, “in silico model”, “virtual patient”), and for their clinical application (“precision medicine”, “drug response prediction”, “ex vivo”), using the Boolean operators AND and OR. No fixed lower date limit was applied, so that seminal earlier work could be included, and the search was last updated in May 2026. Only articles published in English were reviewed; conference abstracts and meeting reports were excluded. Given the breadth of the field, we did not attempt an exhaustive systematic review. We prioritised peer-reviewed primary studies and authoritative reviews on the basis of relevance to the integration of biological avatars and digital twins, methodological rigour, and contribution to the central argument, and we included older, highly cited, or foundational works where they established a concept. Preprints and other non-peer-reviewed sources were not cited. The final reference list was generated on the basis of relevance to the topics covered in this Review.

## Contributors

CLH is the sole contributor. He conceived of the idea, performed the literature searches, synthesised the literature, wrote and edited the manuscript, and created the figures.

## Declaration of interests

CLH receives research support from the Minnesota Partnership for Biotechnology and Medical Genomics.
