# Supplementary material for: Integration of biological avatars and digital twins for “ex vivo clinical trials”
Source: eBioMedicine. 2026 Sep 1;132:106467. doi: 10.1016/j.ebiom.2026.106467 (PMC13570278; doi:10.1016/j.ebiom.2026.106467)
Supplement: Supplementary file 1 [file mmc1.docx]

**Supplementary Tables**

**Supplementary Table 1. Biofabrication and device-integration approaches for biological avatars.**

| **Approach or component** | **Description** | **References** |
| --- | --- | --- |
| Extrusion bioprinting | Bioink expelled from a printhead under pressure, or bioink droplets sprayed onto a substrate | ^1-4^ |
| Lithography bioprinting | Light spatially patterns a cell-laden hydrogel resin | ^5,6^ |
| Bioassembly | Cell spheroids deposited directly onto a predesigned structure without scaffolding | ^7^ |
| Bioinks and resins | Single cells or aggregates suspended in hydrogel for structural support or as sacrificial material; deposited in layers or within a suspension bath | ^8-11^ |
| Hydrogels and matrices | Extracellular-matrix surrogates from Matrigel to engineered polymeric hydrogels tuned for stiffness, ligand density and viscoelasticity | ^12-14^ |
| Microfluidic devices (PDMS) | Biocompatible elastomer shaped by soft lithography into micron-to-millimetre channels, reservoirs and passive pumps; transparency and gas permeability support imaging, low-volume media and air-liquid interfaces; microchannels impose physiological shear stress | ^15-19^ |
| Small-volume and laminar-flow control | Physics of small volumes with laminar flow enabling precise biochemical and shear-stress control at tissue-level interfaces | ^20^ |
| Rapid prototyping | 3D-printing of complex multi-material architectures and incorporation of new hydrogel materials for higher physiological fidelity | ^21-24^ |
| Organ-on-chip exemplars | First lung biomimetic with cyclic stretch; dual-channel adaptations for liver, kidney and intestine; heart and brain chips; fluidically linked 10-organ system | ^25-35^ |
| Material failure modes | PDMS absorption of hydrophobic small molecules distorting pharmacokinetic and pharmacodynamic measurement; mitigated by coatings, thermoplastics or glass, and materials-aware computational correction | ^36-38^ |

Bioprinting modalities, bioink and matrix components, microfluidic device construction, representative organ-on-chip exemplars, and characteristic material failure modes. PDMS, polydimethylsiloxane.

**Supplementary Table 2. Clinically relevant biological avatars by organ system.**

| **Organ system** | **Representative avatar platforms** | **Drugs and processes modelled** | **References** |
| --- | --- | --- | --- |
| Liver | Intrahepatic and extrahepatic cholangiocyte organoids (ICO, ECO); ICO-derived hepatocyte liver-on-chip and cholangiocyte bile-duct-on-chip; vascularised bile-duct-on-chip; iPSC-hepatocyte liver-on-chip (MINERVA 2.0, OrganoPlate, micropatterned coculture, Curiochips); LEADS NASH model | Chlorpromazine-induced bile duct injury in cholestasis; metformin, liraglutide, elafibranor and saroglitazar in oleic-acid steatosis; donepezil transport and toxicity; 159-compound and 47-drug hepatotoxicity screens; acetaminophen necrosis and fialuridine mitochondrial toxicity; NASH treatment response (saroglitazar, pioglitazone, cenicriviroc, obeticholic acid, resmetirom) | ^39-56^ |
| Intestine | Adult-derived enteroids and colonoids; colon-on-chip; 3D tubular scaffold with physiological oxygen gradient; colonoid plus microvascular endothelium with mechanical stretch; iPSC-derived gastric and intestinal organoids; intestine-on-chip | Microbiome-metabolite modulation of enterohaemorrhagic E. coli infection; sustained interaction with human faecal microbiota; IBD-cytokine (IFNgamma, IL22) barrier disruption and IL22BP rescue; drug metabolism (midazolam, diclofenac, S-mephenytoin) and absorption / bioavailability (antipyrine, metoprolol, propranolol, quinidine, atenolol, ranitidine, enalapril, hydrochlorothiazide, sulpiride, erythromycin) | ^57-66^ |
| Lung | Alveolar-capillary interface chip with breathing motion; airway-on-chip from normal, COPD and CF donors; stretchable-membrane and hydrogel chips with microdiaphragm; iPSC-derived lung progenitor seeding | IL-2-induced pulmonary oedema (dose-limiting toxicity); mechanical-strain effects on nanoparticle uptake and silica toxicity; SARS-CoV-2 infection and amodiaquine inhibition (with antimalarials that failed under flow); COPD responses to cigarette smoke; CF mucus, Pseudomonas aeruginosa infection, CXCL8 and neutrophil adhesion | ^25,67-74^ |
| Brain | iPSC- and ESC-derived neural organoids; hindbrain organoids; micropatterned neuroloids; brain organoids with integrated microvasculature, pericytes and astrocytes for a blood-brain barrier | Serotonin-reuptake-inhibitor efficacy screening; high-content screening for rescue of a Huntington disease phenotype; blood-brain barrier modelling for facilitated transport of therapeutics | ^75-78^ |
| Tumour microenvironment | Patient-derived tumoroids incorporating cancer-associated fibroblasts, tumour-infiltrating lymphocytes, tumour-associated macrophages, myeloid-derived suppressor cells and tumour-associated endothelium; adipose microvasculature plus autologous PBMC with lung tumoroids | PD-1 inhibitor response and non-response in non-small-cell lung cancer (PD-L1 shown to be an unreliable stratifier); simultaneous measurement of tumour viability, vascular change and immune activity under chemotherapy and antiangiogenic therapy | ^79,80^ |
| Multi-organ | Fluidically linked body-on-a-chip systems: liver-kidney; reconfigurable liver-heart-tumour; heart-liver-bone-skin; jejunum-liver-kidney-muscle-blood-brain barrier; 7- and 10-organ recirculating systems; laminated vein / artery / organ architecture | Early hepato- and nephrotoxicity from low-dose cyclosporin A; on- and off-target effects of anticancer therapy; doxorubicin PK/PD with early miRNA cardiotoxicity markers; first-pass metabolism (terfenadine to fexofenadine), vitamin D activation, microbiome-metabolite blood-brain barrier penetration; diclofenac and carboplatin pharmacokinetics and clearance | ^35,81-88^ |

Representative platforms and the drugs or processes they have been used to model. COPD, chronic obstructive pulmonary disease; iPSC, induced pluripotent stem cell; NASH, non-alcoholic steatohepatitis; PBMC, peripheral blood mononuclear cell; PK/PD, pharmacokinetic / pharmacodynamic.

**Supplementary Table 3. Comparison of biological avatar platforms.**

| **Avatar platform** | **Principal advantages** | **Principal limitations** | **Suitability for ex vivo clinical trials** | **References** |
| --- | --- | --- | --- | --- |
| Adult stem cell-derived organoids | Patient-specific and genomically stable; rapid to establish from biopsy or resection; retain tissue-specific epithelial architecture and function; near-unlimited proliferation under defined niche factors. | Largely epithelial; cannot capture the full donor cellular repertoire (immune, stromal, neural) without coculture; restricted to tissues with accessible adult stem cells. | Strong for epithelial efficacy and toxicity readouts in bounded, short-window decisions such as colorectal cancer therapy selection and cystic fibrosis modulator testing; weaker where multicellular or systemic interactions dominate. | ^57,59,89^ |
| iPSC-derived organoids | Access to ectodermal, mesodermal and endodermal lineages, including brain, heart and kidney; amenable to genome editing and disease modelling; scalable from a renewable source. | Often functionally immature; longer and more variable derivation; reprogramming and directed differentiation add quality-control burden and batch-to-batch variability. | Useful where adult stem cells are inaccessible or where defined genetic backgrounds are required; maturity and reproducibility currently constrain decision-grade use. | ^75,90,91^ |
| Organ-on-a-chip (single-organ MPS) | Adds perfusion, shear, mechanical actuation and real-time sensing to organoid biology; reproduces organ-level dynamics and barrier function that static culture cannot. | Engineering and operational complexity; polydimethylsiloxane absorbs hydrophobic compounds and can distort pharmacokinetic readouts; lower throughput; reproducibility depends on fabrication and calibration. | Strong where flow, barrier or mechanical context is mechanistically tied to the readout (liver-chip DILI, lung, gut); materials-aware correction needed for quantitative pharmacokinetics. | ^25,34,36^ |
| Multi-organ MPS (body- or human-on-a-chip) | Fluidically links multiple tissues to capture interorgan metabolism, first-pass effects and systemic pharmacokinetics that single-organ formats miss; supports physiologically scaled exposure. | Highest quality-control and reproducibility burden; scaling, common-media compatibility and sensor integration remain unresolved; lowest current standardisation. | Best for systemic exposure and interorgan-toxicity questions; presently research-grade rather than decision-grade. | ^35,86,92^ |
| Bioprinted tissue | Defined spatial placement of cells and matrix into organ-like architectures; tunable geometry and vascular patterning. | Bioink and resolution constraints; reproducibility of complex architectures; maturation and perfusion remain limiting. | Useful for structured or vascularised constructs where architecture is decisive; earliest-stage for decision-grade use. | ^1,8^ |

Suitability reflects the context-of-use framing developed in the main text. DILI, drug-induced liver injury; iPSC, induced pluripotent stem cell; MPS, microphysiological system.

**Supplementary Table 4. Spatial-omics and multiplexed-imaging platforms supporting data-driven digital twins.**

| **Platform** | **Modality and what it measures** | **References** |
| --- | --- | --- |
| Visium / Visium HD | Sequencing-based spatial transcriptomics; transcriptome-wide maps across large tissue areas | ^93,94^ |
| Stereo-seq | Sequencing-based spatial transcriptomics at large area and high resolution | ^95,96^ |
| MERFISH | Imaging-based, subcellular single-molecule RNA mapping | ^97,98^ |
| GeoMx Digital Spatial Profiler | Multiplexed, region-of-interest RNA and protein profiling | ^99^ |
| CosMx Spatial Molecular Imager | Cyclic in situ hybridisation (smFISH-style) transcript imaging | ^100,101^ |
| Xenium In Situ | Padlock-probe in situ chemistry with rolling-circle amplification and iterative probe hybridisation; thousands of transcripts at subcellular resolution | ^102,103^ |
| Imaging mass cytometry | Spatial proteomics by metal-tagged antibody imaging | ^104,105^ |
| Multiplexed ion beam imaging (MIBI) | Spatial proteomics by secondary-ion mass spectrometry | ^106^ |
| Cyclic immunofluorescence (CODEX, COMET) | Iterative staining to visualise dozens of proteins per tissue section | ^107,108^ |

Sequencing-based and imaging-based spatial transcriptomics and spatial proteomics platforms that supply the multimodal inputs for generative digital twins. smFISH, single-molecule fluorescence in situ hybridisation.

**Supplementary Table 5. Generative and agentic artificial-intelligence models in digital twin construction.**

| **Model or framework** | **Role in the avatar-twin loop** | **References** |
| --- | --- | --- |
| GAN architectures (SinGAN, COCO-GAN) | Single-image and coordinate-conditioned generation of tissue-scale morphologies; conditioning via projection discriminators or pix2pix-style concatenation | ^109-112^ |
| Diffusion models (SinDDM, Patch Diffusion) | Stable single-image and patchwise training to synthesise texture-rich biological images | ^113,114^ |
| Spatial-omics-guided image editing (SST-editing, IST-editing) | Edit matched immunofluorescence images guided by spatial gene expression to simulate phenotypic change from single-cell to organism scale | ^115,116^ |
| Generative digital twins | Map gene- and protein-expression to morphology for in silico perturbation; neural generators trained on longitudinal trajectories, retrainable across indications | ^117^ |
| Agentic digital twins | Think-decide-act loops combining Bayesian optimisation and active learning with mechanistic constraints to design the next informative experiment within safety bounds | ^118-120^ |
| Self-driving laboratory paradigm | Sequential closed-loop experimentation, ported from materials science and chemistry to drug testing where assay turnaround permits | ^121,122^ |
| Agent-based mechanistic simulators | Rule-based cell agents (PhysiCell, PhysiBoSS) as the substrate for virtual experiments, orchestrated by an LLM-based planner | ^123,124^ |

Generative architectures, spatial-omics-guided editing methods, and agentic frameworks, with their role in the avatar-twin loop. GAN, generative adversarial network; LLM, large language model.

**Supplementary Table 6. Organoid-to-patient prediction studies.**

| **Disease / setting** | **Avatar platform** | **Intervention(s) tested** | **Key predictive finding** | **References** |
| --- | --- | --- | --- | --- |
| Metastatic CRC - irinotecan (prospective) | Patient-derived tumour organoids | Irinotecan +/- 5-FU | Predicted irinotecan response in >80% of patients; failed for 5-FU + oxaliplatin (assay-condition sensitive) | ^125^ |
| Metastatic GI cancers (n~15) | PDOs | Multiple agents | 100% sensitivity, 93% specificity, 88% PPV, 100% NPV by binary sensitive/resistant classification | ^126^ |
| Stage IV CRC (n>100) | PDOs | Multiple agents | Organoid sensitivity correlated with 16 vs 9-month median PFS; resistance HR 3.4 | ^127^ |
| Rectal cancer - chemoradiation | PDOs | Chemoradiation +/- chemotherapy | Direct correlation between ex vivo response profile and patient PFS across cohorts | ^128-130^ |
| Advanced bowel cancer - WEHI (n=30) | PDOs | Multiple agents | 83% overall accuracy; >90% for identifying non-responding treatments | ^131^ |
| CRC - systematic review (16 studies) | PDOs | Standard of care | Positive/potentially positive predictive power in 15 of 16 standard-care studies; 3 of 4 novel therapy studies positive | ^132^ |
| CRC - meta-analysis (20 studies) | PDOs | Multiple agents | Pooled PPV 68% (95% CI 0.61-0.72), NPV 78% (0.72-0.82) vs 47% empirical response rate | ^133^ |
| mCRC - phase 2 trial (n=34) | PDOs (~15-agent panel) | Multiple agents | 17 of 34 subjects met PFS endpoint at 2 months | ^134^ |
| CF - CFTR modulators | Rectal-biopsy organoids | Ivacaftor, lumacaftor | Accurately predicted individual changes in sweat chloride and ppFEV1 | ^135^ |
| CF - ataluren prediction | Rectal-biopsy organoids | Ataluren | No organoid activity correctly predicted phase III clinical failure before readout | ^136,137^ |
| CF - HIT-CF (n=489) | Rectal-biopsy organoids | CFTR modulator panel | Screened and prioritised modulator therapies across 489 patients | ^138,139^ |
| Dilated cardiomyopathy - LMNA | iPSC-derived endothelial cells + cardiomyocytes | KLF2 agonists; lovastatin identified | Identified lovastatin as rescue agent; confirmed functional improvement in matched patients | ^140^ |
| Ulcerative colitis | Colonic organoids | Tofacitinib (JAK inhibitor) | Organoid sensitivity predicted 24-week clinical response | ^141^ |

Study-by-study results for the implementation examples, including predictive performance metrics and non-cancer use cases. CRC, colorectal cancer; CF, cystic fibrosis; GI, gastrointestinal; HR, hazard ratio; mCRC, metastatic CRC; NPV, negative predictive value; PDO, patient-derived organoid; PFS, progression-free survival; PPV, positive predictive value; ppFEV1, percent predicted forced expiratory volume in 1 second. Additional supporting cancer organoid prediction literature^142-149^.

**Supplementary Table 7. Comparison of the ex vivo clinical trial concept to existing approaches.**

| **Approach** | **Human relevance** | **Iterative wet-dry feedback** | **Patient-specific resolution** | **Basis for moving to in-human testing** | **References** |
| --- | --- | --- | --- | --- | --- |
| Conventional animal-to-human translation | Low; species differences drive poor predictivity and have produced catastrophic first-in-human failures. | None; testing is sequential, not coupled. | None; species and population average. | Regulatory toxicology packages; historically weak predictive validity for efficacy and idiosyncratic toxicity. | ^150,151^ |
| Static avatar screening without computational feedback | High (human-derived), but the readout is a single-time-point snapshot. | None; wet-lab assay with no model coupling. | Yes; per-patient avatar. | Empirical sensitivity and specificity of the assay against the clinical endpoint, without uncertainty propagation. | ^125,126^ |
| Digital-only in silico simulation without wet-lab iteration | Model-dependent; only as good as the calibration data. | None with a living system; updated from external data only. | Yes, if individualised with patient data. | Calibration and validation against held-out clinical data; limited by unmodelled biology. | ^152,153^ |
| Integrated avatar-twin ex vivo clinical trial (this framework) | High; living human-derived avatar coupled to a patient-calibrated twin. | Yes; bidirectional, avatar readouts refine the twin and the twin guides the next experiment. | Yes; patient-matched avatar and twin. | Prespecified context-of-use qualification (sensitivity, specificity, calibration, uncertainty bound) with explicit arbitration and no-call rules (Figure 3). | This review |

Contrasts the integrated avatar-twin ex vivo clinical trial with the main alternatives. Suitability and relevance are expressed relative to context of use rather than as absolute rankings.

**Supplementary Table 8. Illustrative qualification panels for three representative ex vivo clinical trial use cases.**

| **Use case** | **Avatar platform** | **Reference / benchmark panel** | **Primary readout and metric** | **Demonstrated performance (source)** | **Illustrative qualification anchor** | **Principal current limitation** |
| --- | --- | --- | --- | --- | --- | --- |
| Drug-induced liver injury (DILI) | Human liver-on-chip (hepatocyte plus endothelial, Kupffer and stellate cells) | IQ MPS reference compounds: 27 blinded drugs including seven matched toxic / non-toxic structural pairs, ranked by Garside DILI severity | Decreased albumin (IC50), increased ALT, morphology injury score; margin-of-safety figure compared against a threshold value of 50 | 87% sensitivity, 100% specificity across the 27-drug blinded set^154^ | Meets IQ MPS qualification guidelines for the DILI context of use | Captures intrinsic, concentration-dependent injury; does not capture idiosyncratic DILI, which requires immune components the model lacks |
| Colorectal cancer therapy prediction | Patient-derived tumour organoids, with optional stromal or autologous immune coculture | Standard-of-care agents (5-fluorouracil, irinotecan, oxaliplatin) with or without targeted agents | Viability (CellTiter-Glo or CyQUANT); AUC / GRAUC / IC50 with a prespecified sensitivity cutoff (e.g. normalised GRAUC 0.63) | Pooled PPV ~68%, NPV ~78% vs 47% baseline^133^; prospective 5-FU + oxaliplatin AUROC 0.78 to 0.88^155^ | Prespecified sensitivity score plus a minimum AUROC for the specific regimen | Cancer- and regimen-specific; failed for 5-FU plus oxaliplatin in early work; sensitive to assay conditions; evidence dominated by small, often retrospective cohorts |
| Cystic fibrosis modulator testing | Rectal-biopsy-derived intestinal organoids | CFTR modulators (ivacaftor, lumacaftor, tezacaftor, elexacaftor and combinations) | Forskolin-induced swelling; area under the curve at 0.8 µM forskolin | Genotype-level CFTR function r2 ~0.87 vs sweat chloride and modulator-rescue r2 ~0.90 (lung function) / 0.95 (sweat chloride)^156^; FIS associated with long-term FEV1pp decline, n=173^157^ | Prespecified FIS-AUC response magnitude per CFTR genotype class | Ceiling effect limits discrimination among high responders; individual-level prediction weaker than genotype-level association |

Each panel specifies the avatar platform, a published reference / benchmark compound set, the primary readout and quantitative metric, the best demonstrated performance with its source, an illustrative (not consensus) qualification anchor, and the principal current limitation. No field-wide qualification thresholds yet exist; the anchors reflect performance demonstrated to date. ALT, alanine aminotransferase; AUROC, area under the receiver-operating-characteristic curve; CFTR, cystic fibrosis transmembrane conductance regulator; DILI, drug-induced liver injury; FEV1pp, forced expiratory volume in 1 s percent predicted; FIS, forskolin-induced swelling; GRAUC, growth-rate area under the curve; IQ MPS, Innovation and Quality Microphysiological Systems affiliate; NPV, negative predictive value; PPV, positive predictive value.

**Supplementary References**

1.	Ozbolat IT, Hospodiuk M. Current advances and future perspectives in extrusion-based bioprinting. *Biomaterials* 2016; **76**: 321–43.

2.	Li X, Liu B, Pei B, et al. Inkjet Bioprinting of Biomaterials. *Chem Rev* 2020; **120**(19): 10793–833.

3.	Daly AC, Prendergast ME, Hughes AJ, Burdick JA. Bioprinting for the Biologist. *Cell* 2021; **184**(1): 18–32.

4.	Sun W, Starly B, Daly AC, et al. The bioprinting roadmap. *Biofabrication* 2020; **12**(2): 022002.

5.	Lim KS, Levato R, Costa PF, et al. Bio-resin for high resolution lithography-based biofabrication of complex cell-laden constructs. *Biofabrication* 2018; **10**(3): 034101.

6.	Moroni L, Burdick JA, Highley C, et al. Biofabrication strategies for 3D in vitro models and regenerative medicine. *Nat Rev Mater* 2018; **3**(5): 21–37.

7.	Moldovan NI, Hibino N, Nakayama K. Principles of the Kenzan Method for Robotic Cell Spheroid-Based Three-Dimensional Bioprinting<sup/>. *Tissue Eng Part B Rev* 2017; **23**(3): 237–44.

8.	Groll J, Burdick JA, Cho DW, et al. A definition of bioinks and their distinction from biomaterial inks. *Biofabrication* 2018; **11**(1): 013001.

9.	Brunel LG, Hull SM, Heilshorn SC. Engineered assistive materials for 3D bioprinting: support baths and sacrificial inks. *Biofabrication* 2022; **14**(3).

10.	Kang HW, Lee SJ, Ko IK, Kengla C, Yoo JJ, Atala A. A 3D bioprinting system to produce human-scale tissue constructs with structural integrity. *Nat Biotechnol* 2016; **34**(3): 312–9.

11.	McCormack A, Highley CB, Leslie NR, Melchels FPW. 3D Printing in Suspension Baths: Keeping the Promises of Bioprinting Afloat. *Trends Biotechnol* 2020; **38**(6): 584–93.

12.	Xu R, Ooi HS, Bian L, Ouyang L, Sun W. Dynamic hydrogels for biofabrication: A review. *Biomaterials* 2025; **320**: 123266.

13.	Zhao KY, Du YX, Cao HM, Su LY, Su XL, Li X. The biological macromolecules constructed Matrigel for cultured organoids in biomedical and tissue engineering. *Colloids Surf B Biointerfaces* 2025; **247**: 114435.

14.	Huang X, Wang Y, Wang T, Wen F, Liu S, Oudeng G. Recent advances in engineering hydrogels for niche biomimicking and hematopoietic stem cell culturing. *Front Bioeng Biotechnol* 2022; **10**: 1049965.

15.	Zhao Y, Wang EY, Lai FBL, Cheung K, Radisic M. Organs-on-a-chip: a union of tissue engineering and microfabrication. *Trends Biotechnol* 2023; **41**(3): 410–24.

16.	Raj M K, Chakraborty S. PDMS microfluidics: A mini review. *Journal of Applied Polymer Science* 2020; **137**(27): 48958.

17.	Scott SM, Ali Z. Fabrication Methods for Microfluidic Devices: An Overview. *Micromachines (Basel)* 2021; **12**(3).

18.	Xu J, Harasek M, Gfohler M. From Soft Lithography to 3D Printing: Current Status and Future of Microfluidic Device Fabrication. *Polymers (Basel)* 2025; **17**(4).

19.	Inglebert M, Locatelli L, Tsvirkun D, et al. The effect of shear stress reduction on endothelial cells: A microfluidic study of the actin cytoskeleton. *Biomicrofluidics* 2020; **14**(2): 024115.

20.	Christov IC. Soft hydraulics: from Newtonian to complex fluid flows through compliant conduits. *J Phys Condens Matter* 2021; **34**(6).

21.	Juang YJ, Chiu YJ. Fabrication of Polymer Microfluidics: An Overview. *Polymers (Basel)* 2022; **14**(10).

22.	Chen C, Mehl BT, Munshi AS, Townsend AD, Spence DM, Martin RS. 3D-printed Microfluidic Devices: Fabrication, Advantages and Limitations-a Mini Review. *Anal Methods* 2016; **8**(31): 6005–12.

23.	Salentijn GI, Oomen PE, Grajewski M, Verpoorte E. Fused Deposition Modeling 3D Printing for (Bio)analytical Device Fabrication: Procedures, Materials, and Applications. *Anal Chem* 2017; **89**(13): 7053–61.

24.	Gnatowski P, Ansariaghmiuni M, Pilat E, et al. Hydrogel membranes in organ-on-a-chip devices: A review. *Colloids Surf B Biointerfaces* 2025; **251**: 114591.

25.	Huh D, Matthews BD, Mammoto A, Montoya-Zavala M, Hsin HY, Ingber DE. Reconstituting organ-level lung functions on a chip. *Science* 2010; **328**(5986): 1662–8.

26.	Vernetti LA, Senutovitch N, Boltz R, et al. A human liver microphysiology platform for investigating physiology, drug safety, and disease models. *Exp Biol Med (Maywood)* 2016; **241**(1): 101–14.

27.	Jang KJ, Mehr AP, Hamilton GA, et al. Human kidney proximal tubule-on-a-chip for drug transport and nephrotoxicity assessment. *Integr Biol (Camb)* 2013; **5**(9): 1119–29.

28.	Kim HJ, Huh D, Hamilton G, Ingber DE. Human gut-on-a-chip inhabited by microbial flora that experiences intestinal peristalsis-like motions and flow. *Lab Chip* 2012; **12**(12): 2165–74.

29.	Agarwal A, Goss JA, Cho A, McCain ML, Parker KK. Microfluidic heart on a chip for higher throughput pharmacological studies. *Lab Chip* 2013; **13**(18): 3599–608.

30.	Marsano A, Conficconi C, Lemme M, et al. Beating heart on a chip: a novel microfluidic platform to generate functional 3D cardiac microtissues. *Lab Chip* 2016; **16**(3): 599–610.

31.	Vatine GD, Barrile R, Workman MJ, et al. Human iPSC-Derived Blood-Brain Barrier Chips Enable Disease Modeling and Personalized Medicine Applications. *Cell Stem Cell* 2019; **24**(6): 995–1005 e6.

32.	Park TE, Mustafaoglu N, Herland A, et al. Hypoxia-enhanced Blood-Brain Barrier Chip recapitulates human barrier function and shuttling of drugs and antibodies. *Nat Commun* 2019; **10**(1): 2621.

33.	Pediaditakis I, Kodella KR, Manatakis DV, et al. Modeling alpha-synuclein pathology in a human brain-chip to assess blood-brain barrier disruption. *Nat Commun* 2021; **12**(1): 5907.

34.	Ingber DE. Human organs-on-chips for disease modelling, drug development and personalized medicine. *Nat Rev Genet* 2022; **23**(8): 467–91.

35.	Edington CD, Chen WLK, Geishecker E, et al. Interconnected Microphysiological Systems for Quantitative Biology and Pharmacology Studies. *Sci Rep* 2018; **8**(1): 4530.

36.	Grant J, Ozkan A, Oh C, Mahajan G, Prantil-Baun R, Ingber DE. Simulating drug concentrations in PDMS microfluidic organ chips. *Lab Chip* 2021; **21**(18): 3509–19.

37.	van Meer BJ, de Vries H, Firth KSA, et al. Small molecule absorption by PDMS in the context of drug response bioassays. *Biochem Biophys Res Commun* 2017; **482**(2): 323–8.

38.	Toepke MW, Beebe DJ. PDMS absorption of small molecules and consequences in microfluidic applications. *Lab Chip* 2006; **6**(12): 1484–6.

39.	Banales JM, Huebert RC, Karlsen T, Strazzabosco M, LaRusso NF, Gores GJ. Cholangiocyte pathobiology. *Nat Rev Gastroenterol Hepatol* 2019; **16**(5): 269–81.

40.	Lazaridis KN, LaRusso NF. The Cholangiopathies. *Mayo Clin Proc* 2015; **90**(6): 791–800.

41.	Huch M, Gehart H, van Boxtel R, et al. Long-term culture of genome-stable bipotent stem cells from adult human liver. *Cell* 2015; **160**(1-2): 299–312.

42.	Marsee A, Roos FJM, Verstegen MMA, et al. Building consensus on definition and nomenclature of hepatic, pancreatic, and biliary organoids. *Cell Stem Cell* 2021; **28**(5): 816–32.

43.	Schneeberger K, Sanchez-Romero N, Ye S, et al. Large-Scale Production of LGR5-Positive Bipotential Human Liver Stem Cells. *Hepatology* 2020; **72**(1): 257–70.

44.	Chen C, Jochems PGM, Salz L, et al. Bioengineered bile ducts recapitulate key cholangiocyte functions. *Biofabrication* 2018; **10**(3): 034103.

45.	Verstegen MMA, Roos FJM, Burka K, et al. Human extrahepatic and intrahepatic cholangiocyte organoids show region-specific differentiation potential and model cystic fibrosis-related bile duct disease. *Sci Rep* 2020; **10**(1): 21900.

46.	Myszczyszyn A, Muench A, Lehmann V, et al. A hollow fiber membrane-based liver organoid-on-a-chip model for examining drug metabolism and transport. *Biofabrication* 2025; **17**(2).

47.	Wang Z, Faria J, van der Laan LJW, Penning LC, Masereeuw R, Spee B. Human Cholangiocytes Form a Polarized and Functional Bile Duct on Hollow Fiber Membranes. *Front Bioeng Biotechnol* 2022; **10**: 868857.

48.	Du Y, de Jong IEM, Gupta K, et al. Human vascularized bile duct-on-a chip: a multi-cellular micro-physiological system for studying cholestatic liver disease. *Biofabrication* 2023; **16**(1).

49.	Fanizza F, Boeri L, Donnaloja F, et al. Development of an Induced Pluripotent Stem Cell-Based Liver-on-a-Chip Assessed with an Alzheimer's Disease Drug. *ACS Biomater Sci Eng* 2023; **9**(7): 4415–30.

50.	Bircsak KM, DeBiasio R, Miedel M, et al. A 3D microfluidic liver model for high throughput compound toxicity screening in the OrganoPlate(R). *Toxicology* 2021; **450**: 152667.

51.	Ware BR, Berger DR, Khetani SR. Prediction of Drug-Induced Liver Injury in Micropatterned Co-cultures Containing iPSC-Derived Human Hepatocytes. *Toxicol Sci* 2015; **145**(2): 252–62.

52.	Kim J, Song Y, Jolly AL, et al. High-Throughput Microfluidic 3D Outer Blood-Retinal Barrier Model in a 96-Well Format: Analysis of Cellular Interactions and Barrier Function in Retinal Health and Disease. *Advanced Materials Technologies* 2024; **9**(22): 2400634.

53.	Meyer SR, Zhang CJ, Garcia MA, et al. A High-Throughput Microphysiological Liver Chip System to Model Drug-Induced Liver Injury Using Human Liver Organoids. *Gastro Hep Adv* 2024; **3**(8): 1045–53.

54.	Wang Z, Xing C, van der Laan LJW, Verstegen MMA, Spee B, Masereeuw R. Cholangiocyte organoids to study drug-induced injury. *Stem Cell Res Ther* 2024; **15**(1): 78.

55.	Zhu L, Liu S, Wang Z, et al. Modeling hepatic steatosis with human adult stem cell-derived liver organoids. *iScience* 2025; **28**(5): 112344.

56.	Balachander GM, Ng IC, Pai RR, et al. LEADS - a comprehensive human liver-on-a-chip for non-alcoholic steatohepatitis (NASH) drug testing. *Lab Chip* 2025; **25**(14): 3444–66.

57.	Sato T, Vries RG, Snippert HJ, et al. Single Lgr5 stem cells build crypt-villus structures in vitro without a mesenchymal niche. *Nature* 2009; **459**(7244): 262–5.

58.	Jung P, Sato T, Merlos-Suarez A, et al. Isolation and in vitro expansion of human colonic stem cells. *Nat Med* 2011; **17**(10): 1225–7.

59.	Clevers H. Modeling Development and Disease with Organoids. *Cell* 2016; **165**(7): 1586–97.

60.	Tovaglieri A, Sontheimer-Phelps A, Geirnaert A, et al. Species-specific enhancement of enterohemorrhagic E. coli pathogenesis mediated by microbiome metabolites. *Microbiome* 2019; **7**(1): 43.

61.	Chen Y, Rudolph SE, Longo BN, et al. Bioengineered 3D Tissue Model of Intestine Epithelium with Oxygen Gradients to Sustain Human Gut Microbiome. *Adv Healthc Mater* 2022; **11**(16): e2200447.

62.	Apostolou A, Panchakshari RA, Banerjee A, et al. A Novel Microphysiological Colon Platform to Decipher Mechanisms Driving Human Intestinal Permeability. *Cell Mol Gastroenterol Hepatol* 2021; **12**(5): 1719–41.

63.	Broda TR, McCracken KW, Wells JM. Generation of human antral and fundic gastric organoids from pluripotent stem cells. *Nat Protoc* 2019; **14**(1): 28–50.

64.	McCracken KW, Howell JC, Wells JM, Spence JR. Generating human intestinal tissue from pluripotent stem cells in vitro. *Nat Protoc* 2011; **6**(12): 1920–8.

65.	Workman MJ, Gleeson JP, Troisi EJ, et al. Enhanced Utilization of Induced Pluripotent Stem Cell-Derived Human Intestinal Organoids Using Microengineered Chips. *Cell Mol Gastroenterol Hepatol* 2018; **5**(4): 669–77 e2.

66.	Saito T, Amako J, Watanabe T, Shiraki N, Kume S. Human pluripotent stem cell-derived intestinal organoids for pharmacokinetic studies. *Eur J Cell Biol* 2025; **104**(2): 151489.

67.	Huh D, Leslie DC, Matthews BD, et al. A human disease model of drug toxicity-induced pulmonary edema in a lung-on-a-chip microdevice. *Sci Transl Med* 2012; **4**(159): 159ra47.

68.	Si L, Bai H, Rodas M, et al. A human-airway-on-a-chip for the rapid identification of candidate antiviral therapeutics and prophylactics. *Nat Biomed Eng* 2021; **5**(8): 815–29.

69.	Benam KH, Novak R, Nawroth J, et al. Matched-Comparative Modeling of Normal and Diseased Human Airway Responses Using a Microengineered Breathing Lung Chip. *Cell Syst* 2016; **3**(5): 456–66 e4.

70.	Plebani R, Potla R, Soong M, et al. Modeling pulmonary cystic fibrosis in a human lung airway-on-a-chip. *J Cyst Fibros* 2022; **21**(4): 606–15.

71.	Zamprogno P, Wuthrich S, Achenbach S, et al. Second-generation lung-on-a-chip with an array of stretchable alveoli made with a biological membrane. *Commun Biol* 2021; **4**(1): 168.

72.	Humayun M, Chow CW, Young EWK. Microfluidic lung airway-on-a-chip with arrayable suspended gels for studying epithelial and smooth muscle cell interactions. *Lab Chip* 2018; **18**(9): 1298–309.

73.	Stucki JD, Hobi N, Galimov A, et al. Medium throughput breathing human primary cell alveolus-on-chip model. *Sci Rep* 2018; **8**(1): 14359.

74.	Yadav S, Fujimoto K, Takenaga T, et al. Isogenic induced-pluripotent-stem-cell-derived airway- and alveolus-on-chip models reveal specific innate immune responses. *Nat Biomed Eng* 2025.

75.	Lancaster MA, Renner M, Martin CA, et al. Cerebral organoids model human brain development and microcephaly. *Nature* 2013; **501**(7467): 373–9.

76.	Zivko C, Sagar R, Xydia A, et al. iPSC-derived hindbrain organoids to evaluate escitalopram oxalate treatment responses targeting neuropsychiatric symptoms in Alzheimer's disease. *Mol Psychiatry* 2024; **29**(11): 3644–52.

77.	Metzger JJ, Pereda C, Adhikari A, et al. Deep-learning analysis of micropattern-based organoids enables high-throughput drug screening of Huntington's disease models. *Cell Rep Methods* 2022; **2**(9): 100297.

78.	Kistemaker L, van Bodegraven EJ, de Vries HE, Hol EM. Vascularized human brain organoids: current possibilities and prospects. *Trends Biotechnol* 2025; **43**(6): 1275–85.

79.	Veith I, Nurmik M, Mencattini A, et al. Assessing personalized responses to anti-PD-1 treatment using patient-derived lung tumor-on-chip. *Cell Rep Med* 2024; **5**(5): 101549.

80.	Le H, Deforges J, Hua G, et al. In vitro vascularized immunocompetent patient-derived model to test cancer therapies. *iScience* 2023; **26**(10): 108094.

81.	Brandauer K, Schweinitzer S, Lorenz A, et al. Advances of dual-organ and multi-organ systems for gut, lung, skin and liver models in absorption and metabolism studies. *Lab Chip* 2025; **25**(6): 1384–403.

82.	Juguilon C, Khosravi R, Radisic M, Wu JC. In Vitro Modeling of Interorgan Crosstalk: Multi-Organ-on-a-Chip for Studying Cardiovascular-Kidney-Metabolic Syndrome. *Circ Res* 2025; **136**(11): 1476–93.

83.	Abokor FA, Al Yazeedi S, Baher JZ, Cheung C, Sin DD, Osei ET. Exploring Multi-Organ Crosstalk via the TissUse HUMIMIC Chip System: Lessons Learnt So Far. *Biotechnol Bioeng* 2025.

84.	Huang Q, Yang T, Song Y, et al. A three-dimensional (3D) liver-kidney on a chip with a biomimicking circulating system for drug safety evaluation. *Lab Chip* 2024; **24**(6): 1715–26.

85.	McAleer CW, Long CJ, Elbrecht D, et al. Multi-organ system for the evaluation of efficacy and off-target toxicity of anticancer therapeutics. *Sci Transl Med* 2019; **11**(497).

86.	Ronaldson-Bouchard K, Teles D, Yeager K, et al. A multi-organ chip with matured tissue niches linked by vascular flow. *Nat Biomed Eng* 2022; **6**(4): 351–71.

87.	Vernetti L, Gough A, Baetz N, et al. Functional Coupling of Human Microphysiology Systems: Intestine, Liver, Kidney Proximal Tubule, Blood-Brain Barrier and Skeletal Muscle. *Sci Rep* 2017; **7**: 42296.

88.	Wang J, Zhang H, Qu Y, et al. An eighteen-organ microphysiological system coupling a vascular network and excretion system for drug discovery. *Microsyst Nanoeng* 2025; **11**(1): 89.

89.	Hentschel V, Seufferlein T, Armacki M. Intestinal organoids in coculture: redefining the boundaries of gut mucosa ex vivo modeling. *Am J Physiol Gastrointest Liver Physiol* 2021; **321**(6): G693–G704.

90.	Takahashi K, Tanabe K, Ohnuki M, et al. Induction of pluripotent stem cells from adult human fibroblasts by defined factors. *Cell* 2007; **131**(5): 861–72.

91.	Spence JR, Mayhew CN, Rankin SA, et al. Directed differentiation of human pluripotent stem cells into intestinal tissue in vitro. *Nature* 2011; **470**(7332): 105–9.

92.	Marx U, Beken S, Chen Z, et al. Biology-inspired dynamic microphysiological system approaches to revolutionize basic research, healthcare and animal welfare. *ALTEX* 2025; **42**(2): 204–23.

93.	Maynard KR, Collado-Torres L, Weber LM, et al. Transcriptome-scale spatial gene expression in the human dorsolateral prefrontal cortex. *Nat Neurosci* 2021; **24**(3): 425–36.

94.	Kamel M, Song Y, Solbas A, et al. ENACT: End-to-End Analysis of Visium High Definition (HD) Data. *Bioinformatics* 2025; **41**(3).

95.	Chen A, Liao S, Cheng M, et al. Spatiotemporal transcriptomic atlas of mouse organogenesis using DNA nanoball-patterned arrays. *Cell* 2022; **185**(10): 1777–92 e21.

96.	Han L, Liu Z, Jing Z, et al. Single-cell spatial transcriptomic atlas of the whole mouse brain. *Neuron* 2025; **113**(13): 2141–60 e9.

97.	Chen KH, Boettiger AN, Moffitt JR, Wang S, Zhuang X. RNA imaging. Spatially resolved, highly multiplexed RNA profiling in single cells. *Science* 2015; **348**(6233): aaa6090.

98.	Qian X, Coleman K, Jiang S, et al. Spatial transcriptomics reveals human cortical layer and area specification. *Nature* 2025; **644**(8075): 153–63.

99.	Zollinger DR, Lingle SE, Sorg K, Beechem JM, Merritt CR. GeoMx RNA Assay: High Multiplex, Digital, Spatial Analysis of RNA in FFPE Tissue. *Methods Mol Biol* 2020; **2148**: 331–45.

100.	He S, Bhatt R, Brown C, et al. High-plex imaging of RNA and proteins at subcellular resolution in fixed tissue by spatial molecular imaging. *Nat Biotechnol* 2022; **40**(12): 1794–806.

101.	Campos J, McMurray JL, Certo M, et al. Spatial transcriptomics elucidates localized immune responses in atherosclerotic coronary artery. *EMBO Mol Med* 2025.

102.	Ma X, Chen P, Wei J, et al. Protocol for Xenium spatial transcriptomics studies using fixed frozen mouse brain sections. *STAR Protoc* 2024; **5**(4): 103420.

103.	Xuanyuan Q, Wu H, Sundaramoorthi H, et al. Multimodal spatial transcriptomic characterization of mouse kidney injury and repair. *Nat Commun* 2025; **16**(1): 7567.

104.	Giesen C, Wang HA, Schapiro D, et al. Highly multiplexed imaging of tumor tissues with subcellular resolution by mass cytometry. *Nat Methods* 2014; **11**(4): 417–22.

105.	Korber A, Anthony IGM, Heeren RMA. Mass Spectrometry Imaging. *Anal Chem* 2025; **97**(29): 15517–49.

106.	Angelo M, Bendall SC, Finck R, et al. Multiplexed ion beam imaging of human breast tumors. *Nat Med* 2014; **20**(4): 436–42.

107.	Goltsev Y, Samusik N, Kennedy-Darling J, et al. Deep Profiling of Mouse Splenic Architecture with CODEX Multiplexed Imaging. *Cell* 2018; **174**(4): 968–81 e15.

108.	Rivest F, Eroglu D, Pelz B, et al. Fully automated sequential immunofluorescence (seqIF) for hyperplex spatial proteomics. *Sci Rep* 2023; **13**(1): 16994.

109.	Waqas A, Bui MM, Glassy EF, et al. Revolutionizing Digital Pathology With the Power of Generative Artificial Intelligence and Foundation Models. *Lab Invest* 2023; **103**(11): 100255.

110.	Shaham TR, Dekel T, Michaeli T. SinGAN: Learning a Generative Model From a Single Natural Image. 2019 IEEE/CVF International Conference on Computer Vision (ICCV): IEEE Computer Society; 2019. p. 4569–79.

111.	Lin CH, Chang C-C, Chen Y-S, Juan D-C, Wei W, Chen H-T. COCO-GAN: Generation by Parts via Conditional Coordinating. 2019 IEEE/CVF International Conference on Computer Vision (ICCV): IEEE Computer Society; 2019. p. 4511–20.

112.	Isola P, Zhu JY, Zhou T, Efros AA. Image-to-Image Translation with Conditional Adversarial Networks. 2017 IEEE Conference on Computer Vision and Pattern Recognition (CVPR); 2017 21–26 July 2017; 2017. p. 5967–76.

113.	Kulikov V, Yadin S, Kleiner M, Michaeli T. SinDDM: a single image denoising diffusion model. Proceedings of the 40th International Conference on Machine Learning. Honolulu, Hawaii, USA: JMLR.org; 2023. p. Article 738.

114.	Zhang B, Li S, Feng G, Qian Z, Zhang X. Patch Diffusion: A General Module for Face Manipulation Detection. *Proceedings of the AAAI Conference on Artificial Intelligence* 2022; **36**(3): 3243–51.

115.	Wu J, Koelzer VH. SST-editing: in silico spatial transcriptomic editing at single-cell resolution. *Bioinformatics* 2024; **40**(3).

116.	Wu J, Berg I, Koelzer V. IST-editing: Infinite spatial transcriptomic editing in a generated gigapixel mouse pup. In: Ninon B, Caroline P, Maria V, et al., editors. Proceedings of The 7nd International Conference on Medical Imaging with Deep Learning. Proceedings of Machine Learning Research: PMLR; 2024. p. 1707––24.

117.	Alsaedi S, Gao X, Gojobori T. Beyond digital twins: the role of foundation models in enhancing the interpretability of multiomics modalities in precision medicine. *FEBS Open Bio* 2025.

118.	McDonald MA, Koscher BA, Canty RB, Zhang J, Ning A, Jensen KF. Bayesian Optimization over Multiple Experimental Fidelities Accelerates Automated Discovery of Drug Molecules. *ACS Cent Sci* 2025; **11**(2): 346–56.

119.	Colliandre L, Muller C. Bayesian Optimization in Drug Discovery. *Methods Mol Biol* 2024; **2716**: 101–36.

120.	Volk AA, Abolhasani M. Performance metrics to unleash the power of self-driving labs in chemistry and materials science. *Nat Commun* 2024; **15**(1): 1378.

121.	Tom G, Schmid SP, Baird SG, et al. Self-Driving Laboratories for Chemistry and Materials Science. *Chemical Reviews* 2024; **124**(16): 9633–732.

122.	Bannigan P, Aldeghi M, Bao Z, Häse F, Aspuru-Guzik A, Allen C. Machine learning directed drug formulation development. *Advanced Drug Delivery Reviews* 2021; **175**: 113806.

123.	Ghaffarizadeh A, Friedman SH, Macklin P. BioFVM: an efficient, parallelized diffusive transport solver for 3-D biological simulations. *Bioinformatics* 2016; **32**(8): 1256–8.

124.	Ghaffarizadeh A, Heiland R, Friedman SH, Mumenthaler SM, Macklin P. PhysiCell: An open source physics-based cell simulator for 3-D multicellular systems. *PLoS Comput Biol* 2018; **14**(2): e1005991.

125.	Ooft SN, Weeber F, Dijkstra KK, et al. Patient-derived organoids can predict response to chemotherapy in metastatic colorectal cancer patients. *Sci Transl Med* 2019; **11**(513).

126.	Vlachogiannis G, Hedayat S, Vatsiou A, et al. Patient-derived organoids model treatment response of metastatic gastrointestinal cancers. *Science* 2018; **359**(6378): 920–6.

127.	Wang T, Tang Y, Pan W, et al. Patient-Derived Tumor Organoids Can Predict the Progression-Free Survival of Patients With Stage IV Colorectal Cancer After Surgery. *Dis Colon Rectum* 2023; **66**(5): 733–43.

128.	Ganesh K, Wu C, O'Rourke KP, et al. A rectal cancer organoid platform to study individual responses to chemoradiation. *Nat Med* 2019; **25**(10): 1607–14.

129.	Yao L, Zao XL, Pan XF, Zhang HG, Wang FJ, Qiao PF. Application of tumoroids derived from advanced colorectal cancer patients to predict individual response to chemotherapy. *J Chemother* 2023; **35**(2): 104–16.

130.	Mo S, Tang P, Luo W, et al. Patient-Derived Organoids from Colorectal Cancer with Paired Liver Metastasis Reveal Tumor Heterogeneity and Predict Response to Chemotherapy. *Adv Sci (Weinh)* 2022; **9**(31): e2204097.

131.	Tan T, Mouradov D, Lee M, et al. Unified framework for patient-derived, tumor-organoid-based predictive testing of standard-of-care therapies in metastatic colorectal cancer. *Cell Rep Med* 2023; **4**(12): 101335.

132.	Su C, Olsen KA, Bond CE, Whitehall VLJ. The Efficacy of Using Patient-Derived Organoids to Predict Treatment Response in Colorectal Cancer. *Cancers (Basel)* 2023; **15**(3).

133.	Sakshaug BC, Folkesson E, Haukaas TH, Visnes T, Flobak A. Systematic review: predictive value of organoids in colorectal cancer. *Sci Rep* 2023; **13**(1): 18124.

134.	Jensen LH, Rogatto SR, Lindebjerg J, et al. Precision medicine applied to metastatic colorectal cancer using tumor-derived organoids and in-vitro sensitivity testing: a phase 2, single-center, open-label, and non-comparative study. *J Exp Clin Cancer Res* 2023; **42**(1): 115.

135.	Berkers G, van Mourik P, Vonk AM, et al. Rectal Organoids Enable Personalized Treatment of Cystic Fibrosis. *Cell Rep* 2019; **26**(7): 1701–8 e3.

136.	Zainal Abidin N, Haq IJ, Gardner AI, Brodlie M. Ataluren in cystic fibrosis: development, clinical studies and where are we now? *Expert Opin Pharmacother* 2017; **18**(13): 1363–71.

137.	Zomer-van Ommen DD, Vijftigschild LA, Kruisselbrink E, et al. Limited premature termination codon suppression by read-through agents in cystic fibrosis intestinal organoids. *J Cyst Fibros* 2016; **15**(2): 158–62.

138.	Bierlaagh MC, van Mourik P, Vonk AM, et al. Centralized intestinal organoid generation is a feasible and safe approach for personalized medicine as demonstrated in the HIT-CF Europe Organoid Study. *J Cyst Fibros* 2024; **23**(4): 703–6.

139.	Kleinfelder K, Melotti P, Hristodor AM, et al. CFTR modulators response of S737F and T465N CFTR variants on patient-derived rectal organoids. *Orphanet J Rare Dis* 2024; **19**(1): 343.

140.	Sayed N, Liu C, Ameen M, et al. Clinical trial in a dish using iPSCs shows lovastatin improves endothelial dysfunction and cellular cross-talk in LMNA cardiomyopathy. *Sci Transl Med* 2020; **12**(554).

141.	Jang KK, Hudesman D, Jones DR, et al. Tofacitinib Uptake by Patient-Derived Intestinal Organoids Predicts Individual Clinical Responsiveness. *Gastroenterology* 2024; **167**(7): 1453–6 e5.

142.	Zhao Y, Li S, Zhu L, et al. Personalized drug screening using patient-derived organoid and its clinical relevance in gastric cancer. *Cell Rep Med* 2024; **5**(7): 101627.

143.	Taverna JA, Hung CN, Williams M, et al. Ex vivo drug testing of patient-derived lung organoids to predict treatment responses for personalized medicine. *Lung Cancer* 2024; **190**: 107533.

144.	Khorsandi D, Yang JW, Foster S, et al. Patient-Derived Organoids as Therapy Screening Platforms in Cancer Patients. *Adv Healthc Mater* 2024; **13**(21): e2302331.

145.	Abbasi J. Patient-Derived Organoids Predict Cancer Treatment Response. *JAMA* 2018; **319**(14): 1427.

146.	Sachs N, de Ligt J, Kopper O, et al. A Living Biobank of Breast Cancer Organoids Captures Disease Heterogeneity. *Cell* 2018; **172**(1-2): 373–86 e10.

147.	Han J, Jeong HJ, Choi J, et al. Bioprinted Patient-Derived Organoid Arrays Capture Intrinsic and Extrinsic Tumor Features for Advanced Personalized Medicine. *Adv Sci (Weinh)* 2025; **12**(20): e2407871.

148.	Bozym DJ, Zheng DX, Revach OY, Aref A, Jenkins RW. Patient-derived organotypic tumor spheroids, tumoroids, and organoids: advancing immunotherapy using state-of-the-art 3D tumor model systems. *Lab Chip* 2025; **25**(13): 3038–59.

149.	Yakavets I, Kheiri S, Cruickshank J, et al. Machine learning-assisted exploration of multidrug-drug administration regimens for organoid arrays. *Sci Adv* 2025; **11**(31): eadt1851.

150.	Hackam DG, Redelmeier DA. Translation of research evidence from animals to humans. *JAMA* 2006; **296**(14): 1731–2.

151.	Suntharalingam G, Perry MR, Ward S, et al. Cytokine storm in a phase 1 trial of the anti-CD28 monoclonal antibody TGN1412. *N Engl J Med* 2006; **355**(10): 1018–28.

152.	De Domenico M, Allegri L, Caldarelli G, et al. Challenges and opportunities for digital twins in precision medicine from a complex systems perspective. *NPJ Digit Med* 2025; **8**(1): 37.

153.	Zhang F, Jia R, Gao H, Wu X, Liu B, Wang H. In Silico Modeling and Simulation to Guide Bioequivalence Testing for Oral Drugs in a Virtual Population. *Clin Pharmacokinet* 2021; **60**(11): 1373–85.

154.	Ewart L, Apostolou A, Briggs SA, et al. Performance assessment and economic analysis of a human Liver-Chip for predictive toxicology. *Commun Med (Lond)* 2022; **2**(1): 154.

155.	Smabers LP, Wensink GE, Verissimo CS, et al. Patient-Derived Organoids Predict Treatment Response in Metastatic Colorectal Cancer. *Clin Cancer Res* 2025; **31**(23): 5015–26.

156.	Dekkers JF, Wiegerinck CL, de Jonge HR, et al. A functional CFTR assay using primary cystic fibrosis intestinal organoids. *Nat Med* 2013; **19**(7): 939–45.

157.	Muilwijk D, de Poel E, van Mourik P, et al. Forskolin-induced organoid swelling is associated with long-term cystic fibrosis disease progression. *Eur Respir J* 2022; **60**(2).
